# Hedgehog-responsive PDGFRa(+) fibroblasts maintain a unique pool of alveolar epithelial progenitor cells during alveologenesis

**DOI:** 10.1016/j.celrep.2022.110608

**Published:** 2022-04-05

**Authors:** Feng Gao, Changgong Li, Soula Danopoulos, Denise Al Alam, Neil Peinado, Sha Webster, Zea Borok, GoleNaz Adeli Kohbodi, Saverio Bellusci, Parviz Minoo

**Affiliations:** 1 Division of Neonatology, Department of Pediatrics, LAC+USC Medical Center and Childrens Hospital Los Angeles, Los Angeles, CA 90033, USA; 2 Hastings Center for Pulmonary Research, Keck School of Medicine of University of Southern California, Los Angeles, CA 90033, USA; 3 Division of Pulmonary, Critical Care and Sleep Medicine, University of California San Diego School of Medicine, San Diego, CA 92093, USA; 4 Lundquist Institute for Biomedical Innovation at Harbor-UCLA Medical Center, Torrance, CA 90502, USA; 5 Universities of Giessen and Marburg Lung Center (UGMLC), Justus-Liebig-University Giessen, German Center for Lung Research (DZL), 35390 Giessen, Germany; 6 Lead contact

## Abstract

The lung alveolus is lined with alveolar type 1 (AT1) and type 2 (AT2) epithelial cells. During alveologenesis, increasing demand associated with expanding alveolar numbers is met by proliferating progenitor AT2s (pAT2). Little information exists regarding the identity of this population and their niche microenvironment. We show that during alveologenesis, Hedgehog-responsive PDGFRa(+) progenitors (also known as SCMFs) are a source of secreted trophic molecules that maintain a unique pAT2 population. SCMFs are in turn maintained by TGFβ signaling. Compound inactivation of *Alk5 TβR2* in SCMFs reduced their numbers and depleted the pAT2 pool without impacting differentiation of daughter cells. In lungs of preterm infants who died with bronchopulmonary dysplasia, *PDGFRa* is reduced and the number of proliferative AT2s is diminished, indicating that an evolutionarily conserved mechanism governs pAT2 behavior during alveologenesis. SCMFs are a transient cell population, active only during alveologenesis, making them a unique stage-specific niche mesodermal cell type in mammalian organs.

## In brief

Much of the information regarding heterogeneity of alveolar epithelial cells in the lung is from adult mice. Gao et al. analyze the complexity of the alveolar epithelial cell population during alveologenesis, a process interrupted in the pathogenesis of the neonatal chronic lung disease bronchopulmonary dysplasia.

## Graphical Abstract

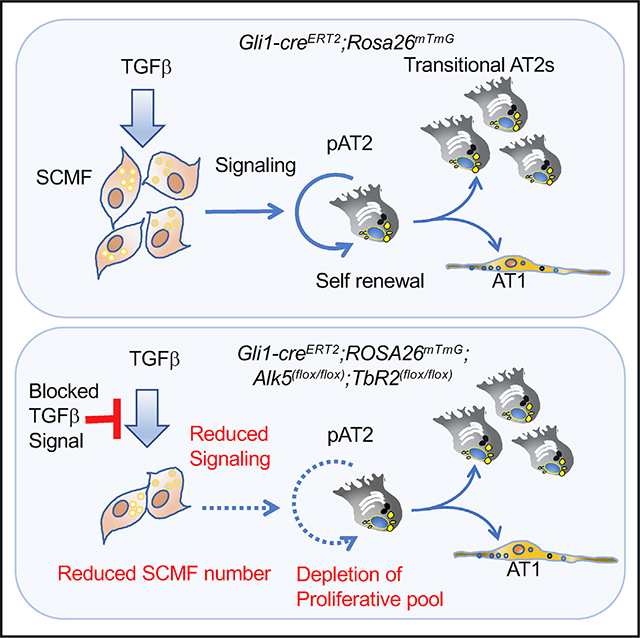

## INTRODUCTION

From the time its primordium is specified within the anterior foregut, lung development is predicated on mesenchymal-epithelial crosstalk (Basil et al., 2020). This communication is particularly complex during alveologenesis, a process that requires concerted functional interactions among multiple cell types. Significant changes in proliferation and differentiation of embryonic cells accompany alveologenesis, which in humans occurs partly and in the mouse entirely postnatally. By completion, the adult alveoli, which number in the millions in mice and man alike, will consist of functional epithelial, mesenchymal, and immune cells, plus a massive capillary plexus, an evolutionary response to oxygen requirement in terrestrial mammals.

The alveolar inner lining is composed of flat alveolar epithelial type 1 cells (AT1s), which are critical for gas exchange, and cuboidal alveolar type 2 cells (AT2s) that produce and secrete pulmonary surfactant. Some AT2s serve as regenerative stem cells (Zacharias et al., 2018; Nabhan et al., 2018). They are key to maintaining homeostasis and repair of lung injury. Regenerative AT2s are WNT responsive and cohabit in niches with specialized mesenchymal cells that direct their behavior. In organoid cultures, platelet-derived growth factor receptor alpha positive (PDGFRa(+)) cells, a heterogeneous population, are thought to serve as mesenchymal “niche” cells (Barkauskas et al., 2013; Zepp et al., 2017). Some candidate mediators of their communication have been identified and functionally tested in organoid cultures (Chung et al., 2018; Green et al., 2016; Zepp et al., 2017). The identity of niche cells *in vivo* and the mechanisms by which they modulate AT2 behavior are less understood. Furthermore, whether temporally specific niches are present during lung development remains unknown.

During alveologenesis, *Sftpc*(+) epithelial progenitors (simply, pAT2s) undergo major expansion in number and differentiate to populate the adult alveoli with AT1s, AT2s, and regenerative AT2s (Frank et al., 2016). The mechanisms are not fully understood. Specifically, whether development of pAT2s is guided within interactive microenvironments akin to the adult niches and the required mechanisms of instructive mesenchymal-epithelial signaling remain unknown. More importantly, whether pAT2s in adult lungs can respond to injuries that occur during alveologenesis is also unknown. These are particularly relevant questions in the context of bronchopulmonary dysplasia (BPD), a chronic lung disease that occurs during alveologenesis in preterm infants.

Alveologenesis requires the formation of “secondary crests” composed of multiple cell types with distinct lineage histories. Located at the tip of secondary crests are PDGFRa(+) cells whose role in alveologenesis has been investigated in a number of studies (Bostrom et al., 2002). A recent study found that 95% of cells lineage-traced with *Pdgfra-rtTA* are located at septal tips in postnatal day 7 (PN7) lungs, cells we previously referred to as secondary crest myofibroblasts (SCMFs) (Li et al., 2018). In the present work we use SCMFs and PDGFRa(+) interchangeably. SCMFs are Hedgehog (HH) responsive and can be targeted effectively by *Gli1-creERT2*, a high-fidelity HH reporter (Ahn and Joyner, 2004; Li et al., 2015). *Pdgfra*^*rtTA*^ also targets SCMFs, and partial ablation of *Pdgra*(+) cells arrests alveologenesis (Li et al., 2018). Despite their designation as “myofibroblasts,” indicating *aSma* expression, SCMFs are distinct from “myofibroblasts,” a term commonly used in reference to pulmonary fibrosis, by quantitatively low *aSma* expression only in the late fetal to early postnatal period and enriched expression of matrix proteins required for alveologenesis (Li et al., 2015). Transforming growth factor β (TGFβ) signaling is increased in SCMFs at the onset of alveologenesis (Li et al., 2015). TGFβ functions by interacting with a heteromeric complex of transmembrane receptors, ALK5 and TβR2. In the lung, TGFβ is commonly associated with lung “injury” and fibrosis, but it also has diverse functions in normal development (Kaartinen et al., 1995; Pieretti et al., 2014).

To determine the significance of TGFβ signaling in SCMFs during alveologenesis and its role in differentiation of AT2 and AT1 cells, we used *Gli1-creERT2* and generated compound mutants with inactivated *Alk5* and *TβR2* in SCMFs, specifically on PN2, at the onset of alveologenesis. The mutations blocked alveologenesis, reducing the number of SCMFs and pAT2s. Single-cell RNA sequencing (RNA-seq) identified a unique population of proliferating pAT2s whose pool was profoundly depleted in the mutant lung. We found that SCMFs constitute a key signaling hub, providing ligands with roles in self-renewal of pAT2s. These characteristics suggest that SCMFs are a principal component of the alveolar niche during alveologenesis. Intriguingly, SCMFs are a transient cell population that are depleted at completion of alveologenesis, making them a unique and temporally specific niche cell type in mammalian organ development (Li et al., 2015; Hagan et al., 2020).

## RESULTS

### Postnatal inactivation of TGFβ receptors in SCMFs arrests alveolar formation, phenocopying human BPD

**Figure 1.**
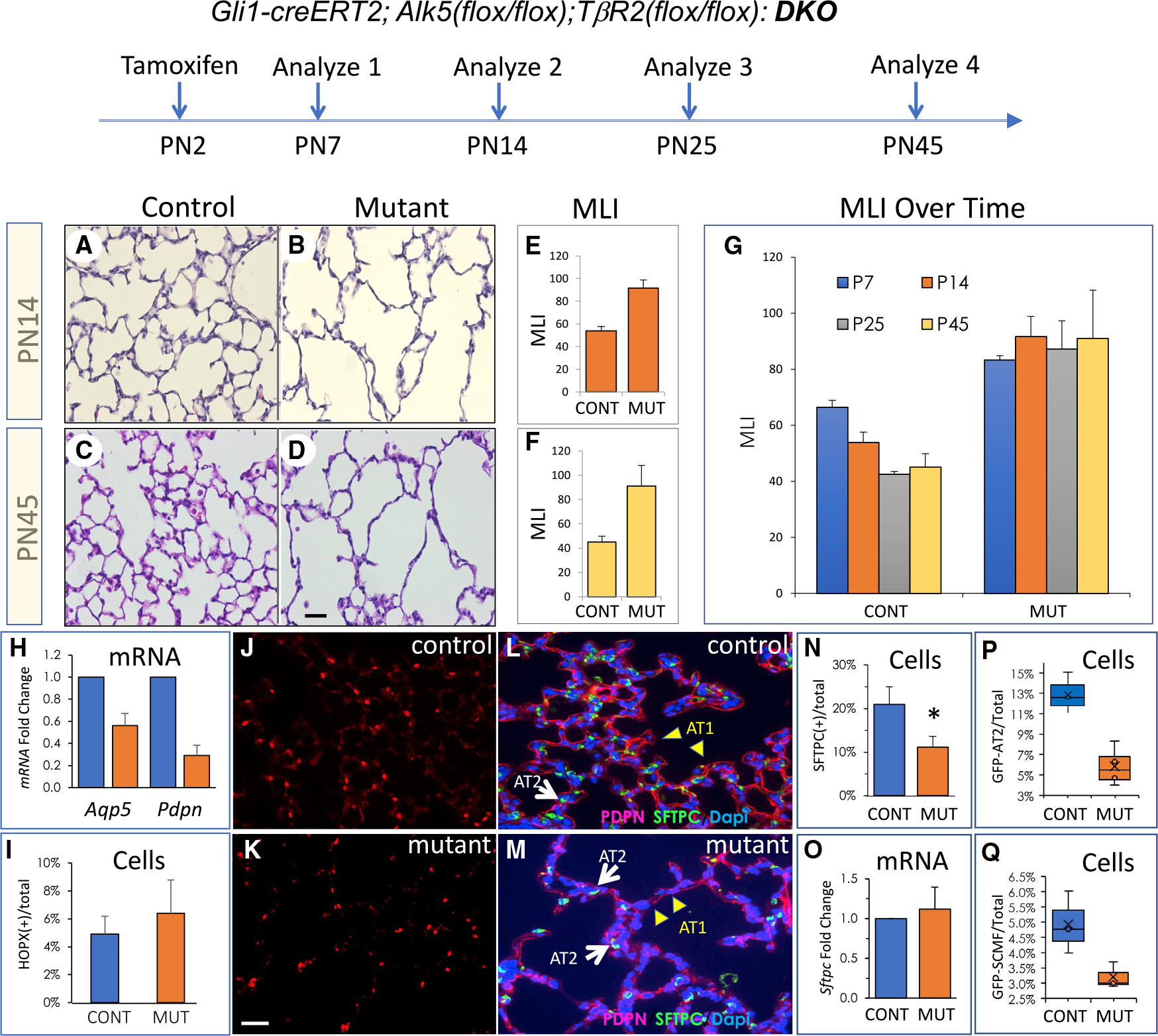
Postnatal inactivation of TGFβ receptors in Hedgehog-responsive SCMFs arrests alveologenesis Newborn *Gli1-creERT2;Alk5*^*(flox/flox)*^*;TbR2*^*(flox/flox*^, (DKO) and control mice received tamoxifen on PN2, and lungs were analyzed between PN7 and PN45. (A–D) Histology of control and mutant lungs at PN14 (A, B) and PN45 (C, D). Scale bar, 50 μm. (E and F) Average of mean linear intercept (MLI) for control versus mutant lungs on PN14 (E) and PN45 (F). (G) MLI of control and mutant lungs over time from PN7 to PN45. (H) Fold change in *Hopx* mRNA between control and mutant lungs. (I) Ratio of HOPX(+) cells to total cells in control versus mutant lungs. (J and K) Immunofluorescent (IF) staining for HOPX (red) using PN14 lung sections from control (J) and mutant (K) mice. (L and M) IF staining for PDPN (red) and SFTPC (green) using PN14 lung sections from control (L) and mutant (M) mice. DAPI (blue) was used to visualize all cells. White arrows point to pAT2s and yellow arrowheads to AT1s. Scale bar, 50um (J&K), 20um (L&M). (N) Ratio of SFTPC(+) cells per total lung cells in control versus mutant lungs. (O) Fold change in *Sftpc* mRNA between control and mutant lungs. (P) FACS-based quantification of GFP-labeled pAT2 cells isolated from *Sftpc-GFP;Alk5*^*(flox/flox)*^*;Tbr2*^*(flox/flox)*^ (control) compared with *Gli1-creERT2;Sftpc-GFP;Alk5*^*(flox/flox)*^;*Tbr2*^*(flox/flox)*^ (mutant). (Q)FACS-based quantification of GFP-labeled SCMFs isolated from *Gli1-creERT2;ROSA26*^*mTmG*^ (control) compared with *Gli1-creERT2;ROSA26*^*mTmG*^*; Alk5*^*(flox/flox)*^*;Tbr2*^*(flox/flox)*^ (mutant). All mice were treated with tamoxifen at PN2 and analyzed on PN14. Data are presented as mean+/−SD (E-I,N,O) and boxplots (P,Q) with n ≥ 3 biological replicates. CONT, control; MUT, mutant.

Both TGFβ receptors were inactivated in *Gli1-cre*^*ERT2*^*; ROSA26*^*mTmG*^*;Alk5*^*(flox/flox)*^*;TβR*^*(flox/flox)*^ newborn mice (here-after DKO) with one dose of tamoxifen on PN2, as described in STAR Methods. We previously showed that this regimen targets SCMFs (Li et al., 2015, 2019). We generated and used *Gli1-cre*^*ERT2*^*;Rosa26*^*mTmG*^ as control. Histology of multiple biological replicates of DKO lungs at two time points, PN14 and PN45, revealed a phenotype of profoundly arrested alveologenesis (alveolar hypoplasia) resembling human BPD lungs (Figures 1A–1D). Quantitative morphometry (n = 3 biological and >10 experimental replicates) using mean linear intercept (MLI) showed increased alveolar size by 52%–58% (Figures 1E and 1F). MLI did not increase in mutant lungs over time, and thus the *DKO* phenotype, unlike other mutations in the TGFβ pathway, is not progressive (Figure 1G). These observations clearly indicate a critical requirement for functional TGFβ receptors in SCMFs during alveologenesis.

### Inactivation of TGFβ receptors in SCMFs reduces both SCMFs and pAT2 cell numbers

Formation of alveoli occurs in close association with specification and maturation of alveolar epithelial progenitors. *Aqp5* and *Pdpn*, both AT1 markers, decreased in *DKO* lungs (Figure 1H). However, AT1 numbers, determined by manual counting of HOPX(+) cells in control and mutant lungs (Figures 1J versus 1K) showed a small difference that failed to reach statistical significance (Figure 1I). In contrast, pAT2 numbers (SFPTC(+)) decreased significantly in the mutant lungs and were not proportional to reduction in total cell numbers, indicating it is not simply a consequence of alveolar hypoplasia (Figures 1L–1N). Changes in *Sftpc* mRNA, if any, did not reach statistical significance (Figure 1O). Reduced pAT2 numbers was confirmed by fluorescence-activated cell sorting (FACS) using GFP-labeled pAT2s (Figure 1P). Total GFP-labeled SCMF numbers also decreased in mutant lungs (Figure 1Q). Neither change was proportional to overall reduction of cell numbers due to hypoplasia. Thus, inactivation of TGFβ receptors in SCMFs results in reduced number of both pAT2s and SCMFs. As adult PDGFRa(+) cells are known to interact with adult AT2s in organoid cultures, we posited that loss of pAT2s in the neonatal lung follows from loss of mutant SCMFs through interruption of SCMFs-pAT2s crosstalk.

### Molecular phenotype of PN14 lungs at single-cell resolution

To elucidate the molecular basis of SCMF-pAT2 crosstalk, we performed single-cell RNA-seq (scRNA-seq) using the 10X Genomic pipeline and determined the molecular phenotype of the entire, intact distal PN14, control (*Gli1-cre*^*ERT2*^*;Rosa26*^*mTmG*^) and mutant (*Gli1-cre*^*ERT2*^*;Rosa26*^*mTmG*^*; Alk5*^*(flox/flox)*^*;Tβr2*^*(flox/flox)*^) lungs at single-cell resolution (STAR Methods). Data were filtered by total unique molecular identifier (UMI), mitochondrial reads, possible doublets, and batch effects, resulting in 12,582 cells from control and 13,819 cells from the mutant PN14 lungs. Median numbers of transcripts per cell were 3,345 and 2,683 for control and mutant lungs, respectively. The median number of genes detected per cell was 1,700 for control and 1,352 for mutant lungs. T-distributed stochastic neighbor embedding (tSNE)-based representation of the combined data revealed four transcriptionally distinct major compartments and 38 cell type subclusters in both genotypes (Figure S1). Well-established cell lineage markers identified the predictive four major compartments, namely mesenchymal or MECs (n = 1,627 expressing *Col1a1*), endothelial or ENCs (n = 6,421 expressing *Cdh5*), immune or IMC (n = 10,723 expressing *Ptprc*), and epithelial or EPC (n = 5,664 expressing *Nkx2.1*) (Figure S2). To focus on the potential genetic changes that might account for altered SCMF-pAT2 cross-communication, we analyzed in detail the scRNA-seq data for MECs and EPCs.

### Identification of the mesenchymal subpopulation disrupted by TGFβ receptor inactivation

**Figure 2.**
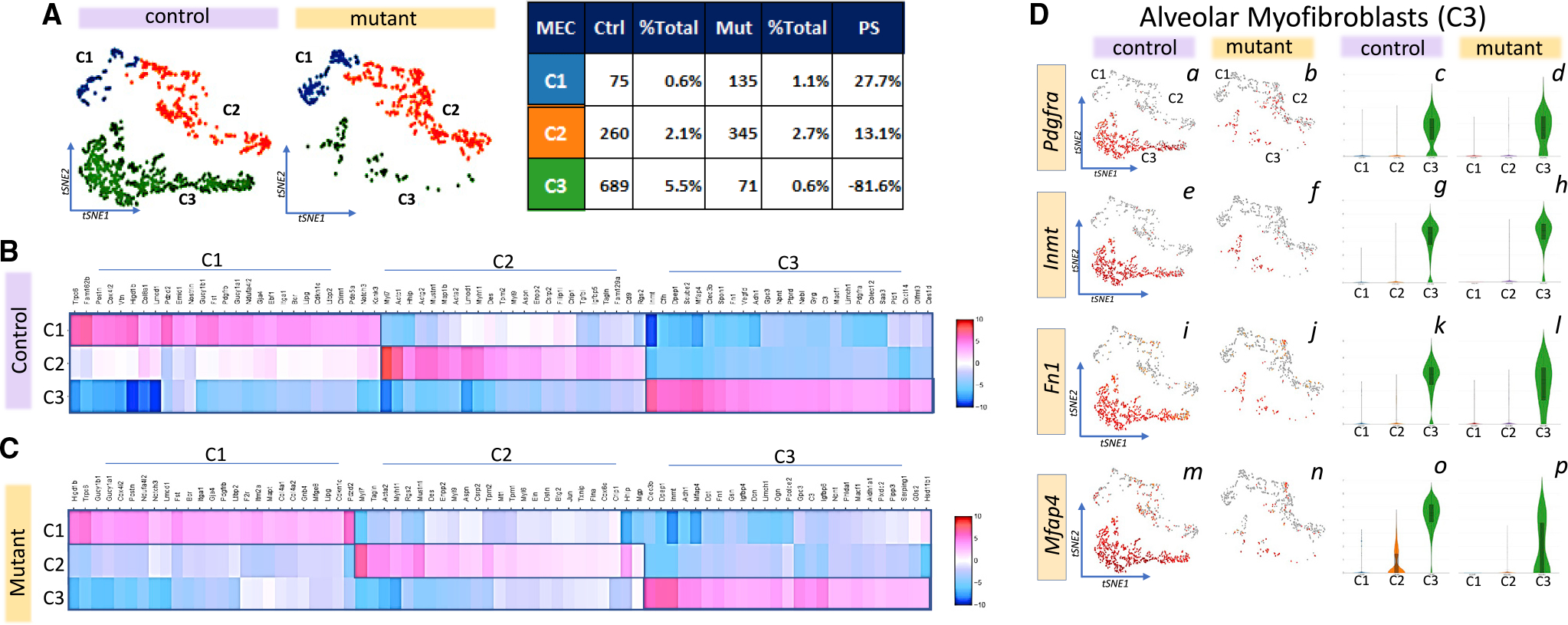
Defining the three distinct mesenchymal subclusters in PN14 lungs (A) tSNE representation of reclustered MECs data revealing three distinct subclusters, C1, C2, and C3, in control versus mutant lungs. The number of cells in each subcluster and their alterations in the mutant lung (PS, population shift) are shown in the table on the right. (B and C) Heatmaps of cell-type-enriched transcripts in each of the three MEC subclusters in control (B) and mutant (C) lungs. (D) tSNE representation of select enriched transcripts for C3, *Pdgfra, Inmt, Fn1*, and *Mfap4*, and violin plot representations of their abundance and differential expression.

Isolation and reclustering of MECs revealed three distinct mesenchymal subclusters C1, C2, and C3 (Figure 2A). Inactivation of TGFβ receptors caused selective loss of C3, contracting it by 81.6% and indicating C3 as the principal target of *Gli1-creERT2* (Figure 2, table). In control lungs, C3 accounts for 67.3% of total MECs and 5.5% of the total number of interstitial cells. These numbers are respectively reduced to 12.9% and 0.6% in the mutant lungs. C3 includes the highest number of mesenchymal cells expressing both *Tbr2* and *Alk5* (Figure S3A) and thus is exquisitely sensitive to loss of TGFβ signaling, which is widely recognized as necessary for growth and maintenance of mesodermal cells.

To assign identity to C3 cells, we analyzed differentially expressed (DE) genes in a three-way comparison among C1, C2, and C3 and determined the enriched transcripts in each of the three subclusters (Figures 2B and 2C) and compared them against signature genes in LungMap (www.lungmap.net). PDGFRA is a widely acknowledged, principal marker of a heterogeneous population of mesenchymal cells found in the alveolar compartment (Chen et al., 2012; Green et al., 2016; McGowan and McCoy, 2014). *Pdgfra* is highly enriched only in C3 (Figure 2D). Lineage traced *Pdgfra*(+) cells are located at the septal tips in PN7 lungs, which we have referred to as SCMFs (Li, et al., 2018). Importantly, *Fn1, Inmt,* and *Mfap4*, which encode extracellular matrix (ECM) proteins required for alveolar formation, are also enriched in C3. C1 and C2 were identified as pericytes and smooth muscle cells, respectively (Figure S3). Computational analysis showed that 80% of the DE genes between control and mutant MECs (i.e., C1 + C2 + C3) are enriched in control C3 and lost in the mutant (Figure S4). Thus, for the larger part, the observed changes in mesenchymal gene expression are reflective of the loss of C3 cells and the pAT2 phenotype in the mutant lungs.

### Identification of a unique proliferative epithelial subpopulation during alveologenesis

**Figure 3.**
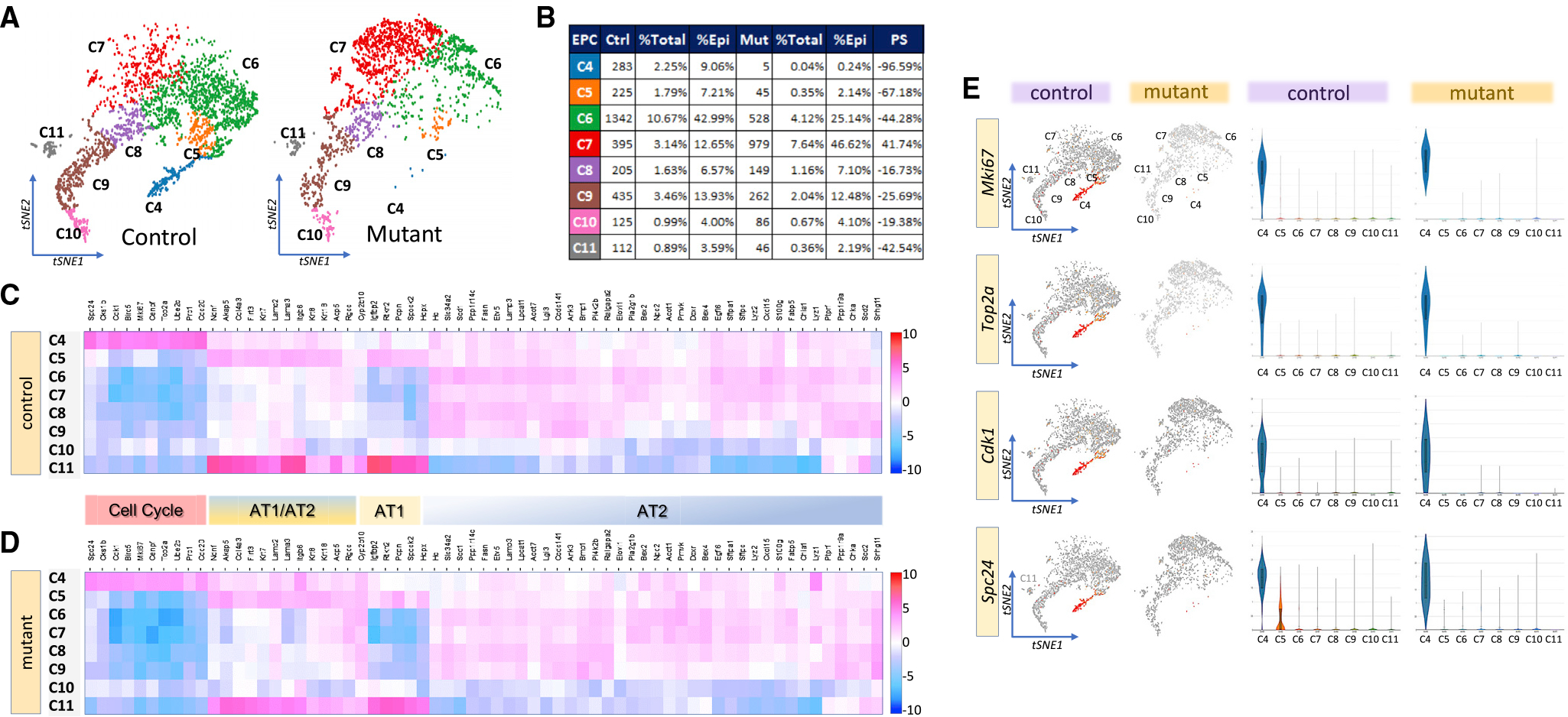
Defining distinct epithelial subclusters in PN14 EPCs (A) tSNE representation of reclustered EPCs data based on *Sfptc* expression, showing eight distinct subclusters, C4 to C11, in both control and mutant lungs. (B) Alterations in cell number in each of the eight subclusters as a fraction of total lung cells, or total epithelial cells between control and mutant lungs. PS, population shift. (C and D) Heatmaps of enriched transcripts in each of the control and mutant subclusters and their overlap with alveolar epithelial cell lineage signature genes derived from LungGens Consortium. (E) tSNE representation of select enriched transcripts for C4 (Mki67, Top2a, Cdk1, and Spc24) and their violin plot abundance and differential expression in C4 to C11.

**Figure 4.**
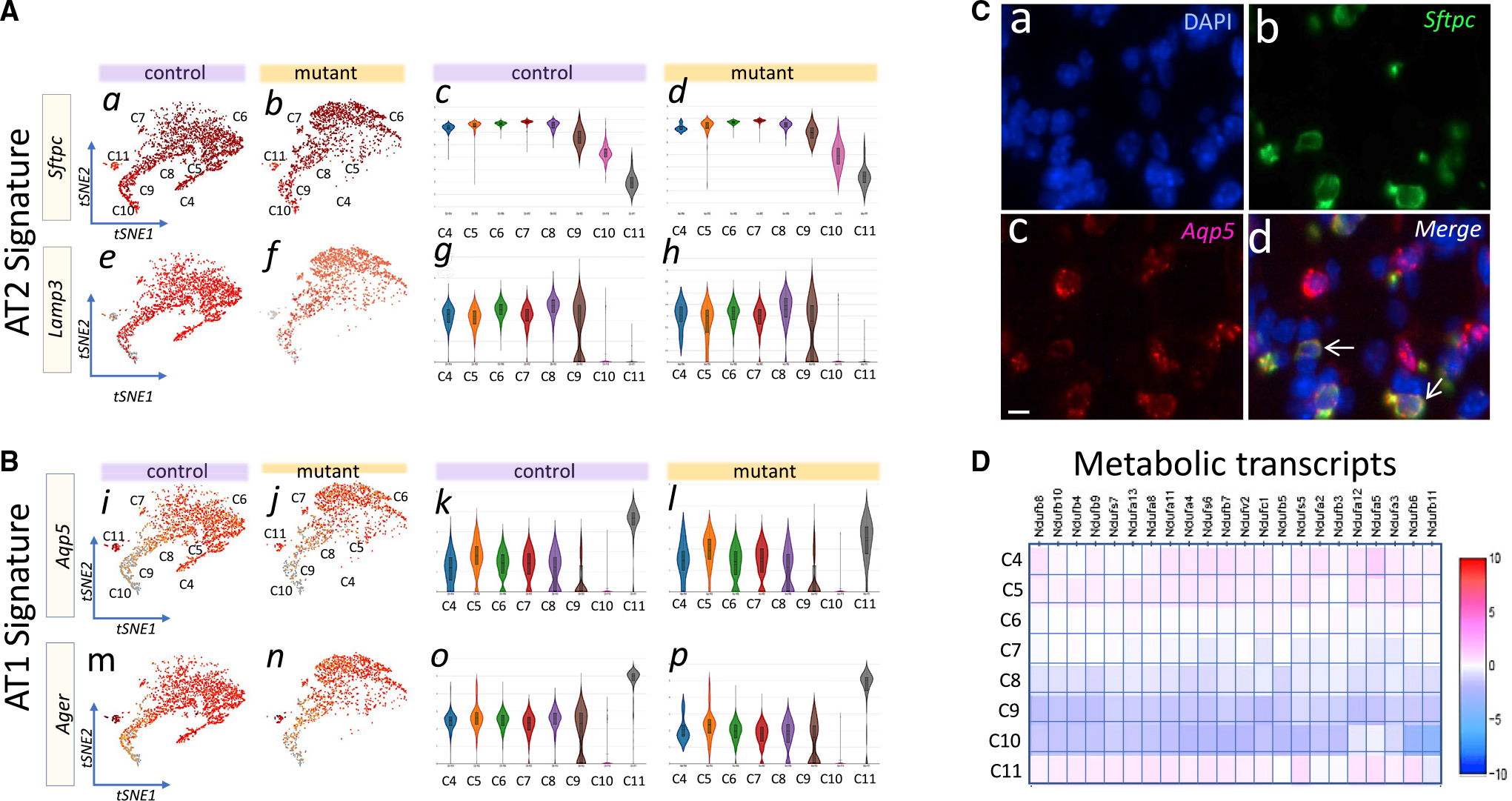
Transitional cells are scattered throughout the epithelial cell population in PN14 lungs (A) tSNE representation of the pAT2 signature gene transcripts *Sftpc* and *Lamp3* in control and mutant epithelial subclusters C4 to C11 (a, b, e, f) and their abundance (c, d, g, h). (B) tSNE representation of the AT1 signature gene transcripts *Aqp5* and *Ager* in control and mutant *Sftpc*(+) subclusters C4 to C11 (i, j, m, n) and their abundance (k, l, o, p). (C) RNAscope using DAPI (a) and probes for *Sftpc* (b), *Aqp5* (c), and their merged images (d) (n = 3). Scale bar, 10 μm. (D) Heatmap of metabolic-related transcripts showing low-level expression of genes in C9 and particularly C10.

Reclustering of the control and mutant EPC data identified eight distinct subpopulations, C4 to C11 (Figure 3A). There were changes in cell numbers in the mutant subclusters (Figure 3B, table). In the control lung, C4 represented 2.25% of total lung cells and 9.06% of total EPCs. These numbers in the mutant lung were reduced to 0.04% and 0.24%, respectively. Thus, a loss of greater than 96% occurred in mutant C4. The identity of cells in C4 to C11 was determined by the extent of overlap between their individual enriched transcripts and the most recent AT1, AT2, and intermediate AT1/AT2 signature genes available on LungMap (www.lungmap.net). C4 represents a unique proliferative and least differentiated subpopulation of pAT2s based on highest expression of cell-cycle genes *Top2a, Mki67, Cdk1,* and *Spc24,* combined with coexpression of low levels of AT1, AT2, and AT1/AT2 markers (Figures 3C–3E). Pathway analysis confirmed enrichment of cell proliferation activity in C4 (Figure S5A). Similar analysis identified C5 to C9 as differentiating pAT2s with progressively increased expression of AT2 signature genes, including lamellar body and pulmonary surfactant genes *Sftpb* and *Sftpa1* (Figures 3C, 3D, and S5B). Importantly, only minor differences exist between the profiles of mutant versus control C5 to C9, indicating no major alterations in lineage or differentiation trajectory in response to loss of TGFβ signaling in SCMFs (Figures 3C versus 3D). However, we were surprised to find that nearly all epithelial clusters displayed coexpression of AT2 and AT1 signature genes (Figures 4A and 4B). Presence of intermediate cells in PN14 lungs was confirmed by RNAscope (Figure 4C). Therefore, C5 through C9 represent intermediate or transitional differentiating pAT2s in PN14 lungs, as late as halfway through the alveologenesis period. Interestingly, in C10 and C11, AT2 markers *Sftpc* and *Sftpb*, and *Sftpa1*, were strongly repressed (Figure S5B). The differences between C10 and C11 are the increased expression of maturing AT1 signature genes *Hopx, Pdpn, Igfbp2,* and *Spock2* (Wang et al., 2018), and metabolic genes in C11. Indeed C11 transcriptome included *Hopx, Pdpn, Igfbp2,* and *Spock2,* consistent with maturing/mature AT1 cells (Figure S7). C10, in contrast, displays a low metabolic profile and reduced AT2 signature genes, indicating a potential inflection point along the differentiation trajectory (Figure 4D).

Pseudo-time trajectory of dynamic gene expression and cell fate transitions suggests that C4 serves as the origin of the transitional epithelial cells described above (Figure S6). Computational analysis of DE genes between control and mutant EPCs, and comparison with enriched genes in each of the EPC subclusters, shows that 85% of the DE genes in the mutant lung originated from C4 (Figure S8). Thus, C4 is the major impacted epithelial population in the mutant lung. As the mutation is exclusively targeted to HH-responsive mesenchymal cells, depletion of the C4 pool likely occurs through disruption of normal SCMFs-pAT2s cross-communication.

### Ligand-receptor (L-R) pairing identifies six major pathways

**Figure 5.**
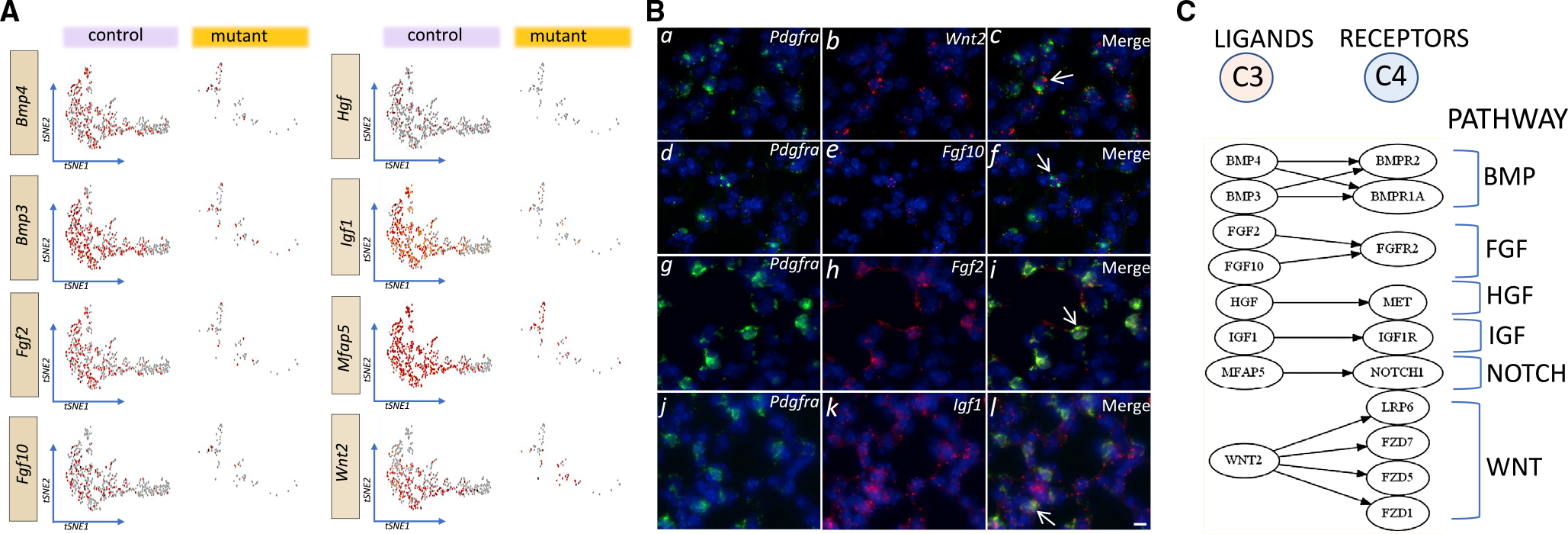
Differentially expressed ligands in C3 and localization of select ligands by RNAscope and ligand-receptor coupling in PN14 lungs (A) tSNE representation of eight identified signaling ligands enriched in C3 and DE (FC > 2 and p ≤ 0.05) between control and mutant lungs. (B) RNAscope analysis of select ligand transcripts, *Wnt2, Fgf10, Fgf2,* and *Igf1,* and their overlap with *Pdgfra* (arrows in c, f, i, and l) in PN14 control lung samples (n = 3). Scale bar, 10 μm. (C) Ligand-receptor coupling diagram and the six principal signaling pathways they represent.

AT2-mesenchymal interactions have been examined largely by *in vitro* or organoid cultures. In the present *in vivo* study, we identified ligands enriched in C3 and differentially expressed between control and mutant lungs by using the computational algorithm shown in Figure S9. Among 251 genes, we identified 13 (fold change [FC] > 2 and p ≤ 0.05) that encoded ligands based on the annotated Fantom Reactome database. *Cp* or ceruloplasmin is expressed in airways and thus may represent contamination in RNA-seq data (Yang et al., 1996). The other 12 genes fell into two functional groups. *Npnt, Cyr61, Bgn,* and *Col6a3* are related to ECM-based signaling. The other eight encode signaling molecules, members of the HGF, FGF, BMP, WNT, and IGF pathways. These are selectively enriched in C3 (Figure 5A). RNAscope revealed overlapping expression of a select number of ligands with *Pdgfra* in PN14 lungs (Figure 5B). Finally, we paired the eight ligand genes in C3 with receptor genes that were defined as actively expressed in pAT2 cells in C4 (i.e., transcripts ≥30 copies/cell) and annotated in our scRNA-seq data using the STAR method (Figure 5C) (Ramilowski et al., 2015). The L-R pairing implicated six major functional pathways, whose reduced signaling, due to SCMF loss in the mutant lung, can account for depletion of the C4 pool (Figure 5C).

### Functional analysis of putative signaling pathways in 3D organoid culture using neonatal pAT2s

**Figure 6.**
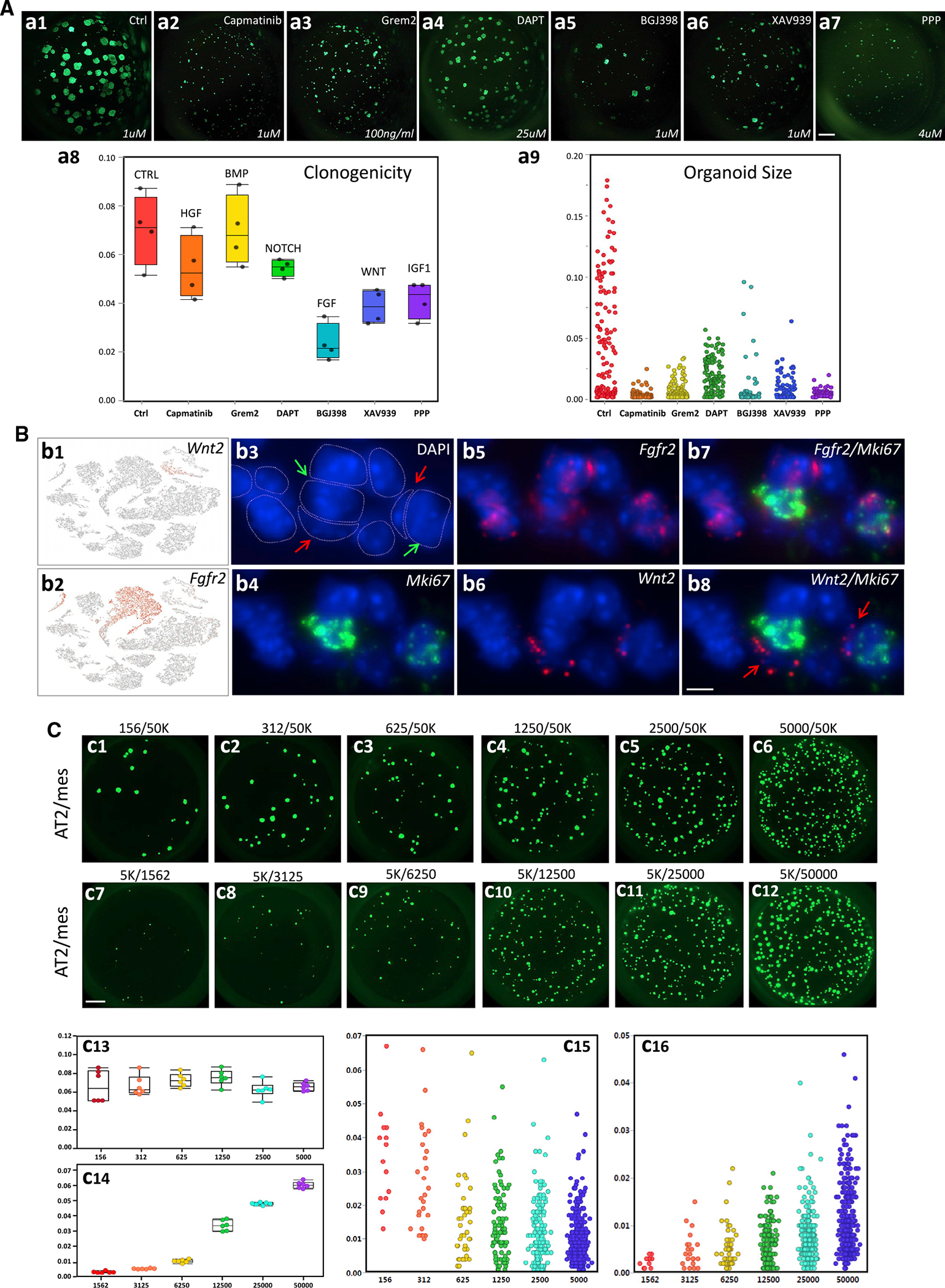
Functional examination of candidate ligands in 3D organoid cultures and localization of Wnt2-expressing cells in PN14 lungs (A) GFP(+) pAT2s were isolated from *Sftpc-Gfp* PN14 lungs and cultured with PN14 mesenchymal cells as described in STAR Methods. 3D cultures were seeded with 1,250 GFP(+) pAT2 cells and 50K mesenchymal cells and were examined on day 28 for direct GFP(+) cells. Ligand-receptor interactions between pAT2 and mesenchymal cells identified in Figure 5 were functionally examined by inhibiting the indicated pathways as shown. Inhibitors were used to block HGF (capmatinib, 1.0 μM), BMP (recombinant GREM2, 100 ng/mL), NOTCH (DAPT, 25 μM), FGF (BDJ398, 1 μM), WNT (XAV939, 1 μM) and IGF1 (PPP, 4 μM). (a1-a7) Photomicrographs of representative organoids in each culture. (a8) quantification of the number of clones (clonogenicity). (a9) Quantification of organoid size. Scale bar, 1mm (a1-a7). (B). (b1 and b2) tSNE representation of *Wnt2* and *Fgfr2* transcript distribution in control lung. (b3–b8) RNAscope analysis showing DAPI alone (b3), Mki67 (b4), *Fgfr2* (b5), *Wnt2* (b6), *Fgfr2/Mki67* merge (b7), and *Wnt2/Mki67* merge (b8). Red arrows show *Wnt2*(+) mesenchymal cells juxtaposed to *Mki67*(+) pAT2 cells, shown by green arrows. Scale bar, 10 μm (b3–b8). (C) Modeling impact of cell population changes on neonatal pAT2 self-renewal. Different ratios of GFP(+) pAT2 cells and mesenchymal cells from PN14 control lungs were cultured as described in STAR Methods. Cell ratios are shown above each panel. 3D cultures were examined on day 21 for direct GFP(+) cells. (c1–c6) Increasing number of GFP(+) pAT2 cells cultured with 50K mesenchymal cells. (c7–c12) 5K GFP(+) pAT2 cells cultured with increasing number of mesenchymal cells as shown. (c13 and c15) Quantification of the number and size of the colonies respectively formed in c1–c6. (c14 and c16) Quantification of the number and size of the colonies, respectively formed in c7–c12. For each culture, n = 3. Scale bar, 1 mm (c1–c12). Data are presented as boxplots with n=4 (a8) and n=6 (c13,c14).

The potential function of the six signaling pathways was examined in *ex vivo* three-dimensional (3D) organoid assays (Barkauskas et al., 2013). FACS-isolated pAT2s from PN14 *Sftpc-GFP* lungs were cocultured with lung mesenchymal cells. In each organoid assay (n = 5 biological replicates) we blocked one of the six principal signaling pathways shown in Figure 6, using specific small-molecule inhibitors for NOTCH, FGF, and IGF1 and recombinant GREM2 for BMP (see Figure 6 legend and Table S1 for concentrations used). The function of individual inhibitors was validated as shown in Figure S10. Blocking HGF with capmatinib, an ATP-competitive inhibitor of c-MET (Clement et al., 2020; Kong et al., 2020; Oh et al., 2021), while dramatically reducing organoid size, had a moderate impact on colony-forming efficiency (CFE) (Figure 6A, a8 and a9). Inhibition of BMP by GREM2 (Tanwar et al., 2014) had no impact on CFE but reduced the organoid size, suggesting its activity may be more related to pAT2 proliferation (Figure 6A, a8 and a9). The most significant inhibition of CFE occurred by blocking FGF using BDJ398 (De Simone et al., 2021; Qiu et al., 2019; van den Brink et al., 2020), but also reduced organoid size (Figure 6A, a8 and a9). Inhibition of NOTCH, WNT, and IGF1, using DAPT (Rognoni et al., 2014), XAV939 (Burridge et al., 2014), and PPP (Gong et al., 2019), respectively (STAR Methods), had similar impact on clonogenicity (Figure 6A, a8). NOTCH inhibition had the most moderate impact and IGF1 and WNT the most profound impact on organoid size (Figure 6A, a9). WNT2 is reported to be associated with self-renewal of stem cells (Zeng and Nusse, 2010), and we found it to be expressed nearly exclusively in C3 (Figure 6B, b1). Using *Fgfr2* as a marker of AT2s in PN14 lungs (LungGens Consortium and Figure 6B, b2) RNAscope showed that *Wnt2*(+) cells are juxtaposed to proliferating, Mki67;*Fgfr2* double-positive cells, indicating WNT2 may be a likely candidate in regulating self-renewal of C4 cells during alveologenesis (Figure 6B, b3–b8). Collectively, the results implicate a rather complex network of multiple signaling pathways, which functionally converge to regulate both clonogenicity and cell proliferation of PN14 pAT2s during postnatal alveologenesis.

### Modeling the impact of mesenchymal cell population changes on neonatal pAT2 self-renewal

In the mutant lung, both SCMFs and pAT2s decreased. To examine the significance of cell number changes (i.e., population shifts), we re-employed the 3D organoid culture combined with variable pAT2/mesenchymal cell stoichiometry using control cells. When cocultured with a fixed number of mesenchymal cells (50K), CFE was independent of pAT2 numbers, indicating that only a limited, constant fraction of pAT2s in each sample is clonogenically competent (i.e., progenitor/stem cell property) (Figure 6C, c1–c6 and c13). However, organoid size, more a measure of pAT2 self-renewal, was inversely proportional to pAT2 numbers (Figure 6C, c15). Conversely, when pAT2 numbers were fixed at 5K, CFE as well as organoid size were directly proportional to the number of cocultured mesenchymal cells (Figure 6C, c7–c12, c14, and c16). Therefore, a threshold level of mesenchymal cell number is required, below which organoid formation is nearly undetectable. Overall, as the mesenchyme is the source of factors required for stimulating organoid formation and growth, the results show that decreasing the signaling amplitude by increasing pAT2 numbers, or decreasing mesenchymal cell number, limits accessibility of individual pAT2s to growth molecules, thus resulting in smaller average size per organoid (i.e., cell numbers). This condition mimics and explains the findings in the TGFβ receptor mutant lungs.

### The mechanism governing pAT2 behavior in alveologenesis is evolutionarily conserved in the human neonatal chronic lung disease BPD

**Figure 7.**
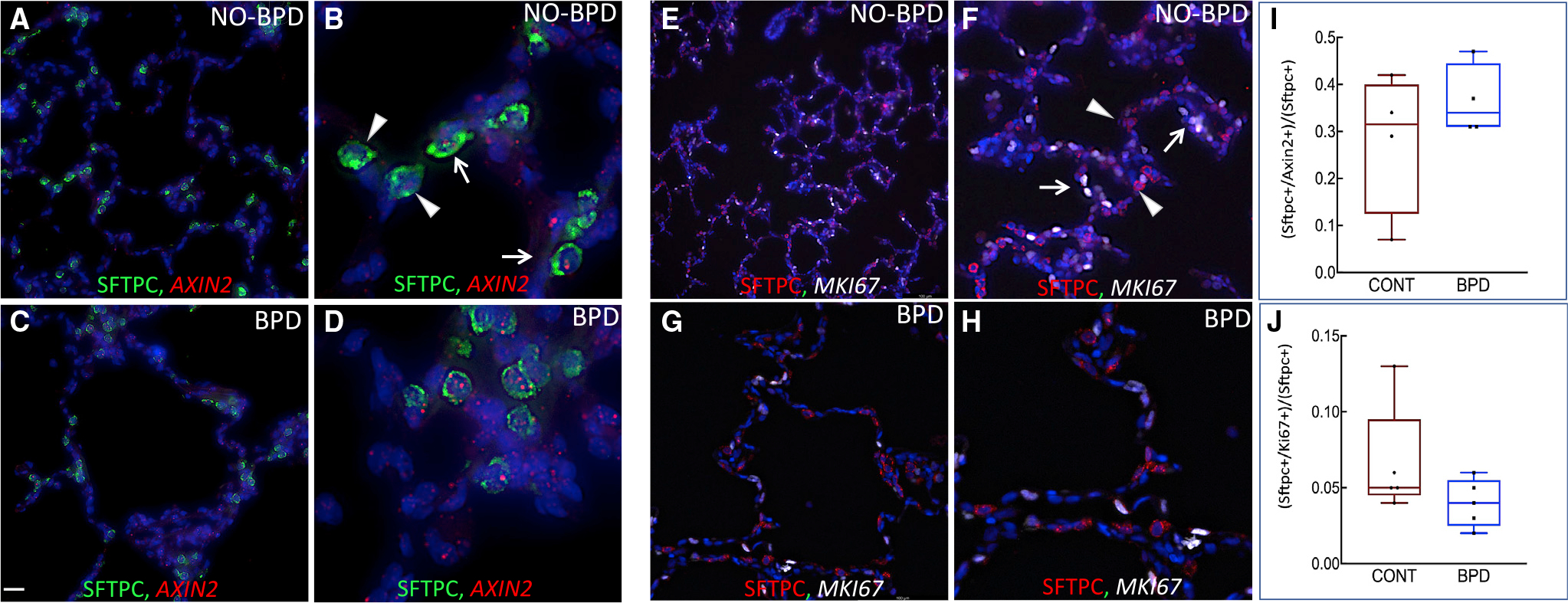
Regenerative pAT2s in human BPD and non-BPD lungs (A–D) Combination IF for SFTPC (green) and RNAscope for *AXIN2* (red) used to identify regenerative SFTPC(+);*AXIN2*(+) double-positive cells. (A) Representative control human lung sample from a full-term neonate who died of non-pulmonary causes. (B) High magnification of lung shown in (A). Arrowheads show SFTPC(+) cells and arrows show SFTPC(+);*AXIN2*(+) double-positive cells. (C) Representative BPD lung sample from a male preterm neonate born at 23 weeks gestation who died with BPD. (D) High magnification of lung shown in (C). (E–H) Combination IF for SFTPC (red) and RNAscope for *MKI67* (white) used to identify and quantify of proliferative AT2s. (E) Representative control human lung sample from a full-term neonate who died of non-pulmonary causes. (F) Higher magnification of the same lung as shown in (E). Arrowheads show SFTPC(+) and arrows show SFTPC(+);*MKI67*(+) double-positive cells. (G) Representative BPD lung from a male preterm neonate born at 24 weeks gestation who died with BPD. (H) High magnification of lung shown in (G). (I) Quantification of the number of SFTPC(+);*AXIN2*(+) double-positive cells as a fraction of total SFTPC(+) cells. (J) Quantification of SFTPC(+);*MKI67*(+) double-positive cells as a fraction of total SFTPC(+) cells. n = 4 biological and 15–20 experimental replicates. Scale bars, 40 μm (A, C, F, H, G), 10 μm (B, D), and 100 μm (E). Data are presented as boxplots with n=4 (I) and n=5 (J).

BPD, which is characterized by alveolar hypoplasia, is a highly complex, variable, and poorly understood neonatal lung disease (Sahni and Mowes, 2020). Two major etiologic factors are lung immaturity and injury. In adult mice, lung injury triggers AT2 self-renewal and differentiation as part of the regenerative/repair mechanism. Mouse *Axin2*(+);*Sftpc*(+) cells, referred to as alveolar epithelial progenitors (AEPs), show enhanced regenerative potential compared with *Sftpc*(+) cells (Zacharias et al., 2018). The adult human lung contains an equivalent of the mouse *Axin2*(+);*Sftpc*(+) cells (Zacharias et al., 2018). Whether such regenerative cells are present in human neonatal lungs and their population size in BPD are unknown. In the present study, loss of *Pdgfra*(+) SCMFs inhibited alveologenesis and depleted the pAT2 pool. Reduced *PDGFRa* is a primary feature of BPD (Popova et al., 2014). However, the size of the proliferative pAT2 pool in BPD lungs is unknown. We used a combination of immunofluorescence (IF) staining and RNAscope to determine the size of the proliferative pAT2 pool and the extent of AT2 proliferation using multiple human BPD and control samples (n = 4 biological replicates, n = 15–20 experimental replicates for each). Quantification of the number of *AXIN2*(+);SFTPC(+) cells as a fraction of total pAT2 cells, which corrects for alveolar hypoplasia, was similar or greater in BPD lungs compared with controls (Figures 7A–7D and 7I). Importantly however, double-positive SFTPC(+);*MKI67*(+) proliferative cells were distinctly reduced in BPD lungs (Figures 7E–7H and 7J). These data suggest that while BPD lungs may contain a larger or similar reservoir of the presumptive Axin2(+)-pAT2 regenerative cells, they appear to lack the normal proliferative response to injury.

## DISCUSSION

We used combination of loss-of-function genetics, scRNA-seq, and 3D organoid cultures to elucidate the role of HH-responsive, *Pdgfra*(+) cells, previously referred to as SCMFs, in alveologenesis. Inactivation of TGFβ receptors in SCMFs caused significant loss of these cells, a principal source of trophic molecules, and arrested alveolar formation, a phenocopy of human BPD. Reduced SCMF numbers profoundly depleted a unique pool of proliferative epithelial cells, which by inference from trajectory mapping serve as a reservoir for differentiating AT1 and AT2 cells. These findings support a model in which TGFβ signaling is critical for proliferation of SCMFs, which in turn, via crosstalk, maintains pAT2 self-renewal. Finally, using human BPD lung samples, we show that as in the mouse model, reduced self-renewal of pAT2s and not their absence may underlie pathogenesis of this neonatal chronic lung disease.

Inactivation of TGFβ receptors in the mutant lungs resulted in 81% asymmetric reduction of SCMFs represented by subcluster C3 (Figure 2). C3 comprises the largest number of *Alk5*(+) and *Tbr2*(+) expressing cells (Figure S3), making their proliferation particularly sensitive to loss of TGFβ signaling, widely recognized for its role in regulation of mesenchymal growth and proliferation (Grafe et al., 2018). Indeed, isolated mutant GFP-labeled SCMF cell lines display reduced proliferation *in vitro* compared with controls (Figure S11). We demonstrate that SCMFs are a mesenchymal signaling hub for a suite of trophic paracrine molecules, including ligands for six major signaling networks. The consequences of C3 cell losses may include reduced availability of these ligands to the proliferating and differentiating epithelial progenitor cells that are required during alveologenesis. The mutant lungs also contained significantly fewer pAT2s due to reduced proliferation, as revealed by analysis of proliferating SFPTC(+) cells across PN7 and PN14 control and mutant lungs (Figure S11). Analysis by scRNA-seq showed that the *Sfptc*(+) compartment is composed of eight subpopulations. The most impacted population was C4, comprising the least differentiated, most highly proliferative epithelial progenitors. Loss of TGFβ and the consequent reduction in SCMF numbers depleted the C4 pool by 96% (Figure 3B).

Gene enrichment analysis and pseudo-time trajectory mapping inferred that C4 serves as a reservoir for differentiating alveolar epithelial transitional subclusters (Figure S6). In these subclusters we found widespread presence of epithelial intermediate cells coexpressing mature AT2 (*Sftpc, Fasn*) and multiple AT1 markers (e.g., *Aqp5* and *Ager*). Epithelial cells coexpressing AT1 and AT2 genes, so-called bipotential, have been described in the saccular phase of lung development but are exhausted by PN4 (Desai et al., 2014, Treutlein et al., 2014). Intermediate AT1/AT2 cells were also reported in early PN1 lungs (Guo et al., 2019). The abundant presence of intermediate cells in PN14 may represent plasticity and incomplete cell fate determination as late as the mid-alveologenesis period. Interestingly, intermediate AT2/AT1 cells have also been observed during alveolar regeneration in multiple injury models including lipopolysaccharide (Riemondy et al., 2019) and bleomycin (Kobayashi et al., 2020; Strunz et al., 2020). Thus, it appears that regeneration of injured alveoli may largely recapitulate the developmental process of pAT2s during alveologenesis. It is important to note that in both mutant and control lungs, once the daughter cells exited the C4 pool, their differentiation followed a similar trajectory (Figures 3C and 3D). As SCMFs are a transient cell population depleted at completion of alveologenesis (Li et al., 2015; Hagan et al., 2020), their principal function may be limited to providing trophic factors that stimulate or maintain self-renewal of pAT2s only during alveologenesis.

In general, knowledge of factors regulating AT2 self-renewal *in vivo* and during alveologenesis remains incomplete. In adult lungs, expression of *Fgf7, Bmps*, and *Il6* are reported as potential regulators of AT2 behavior and have been examined in organoid cultures (Chung et al., 2018; Green et al., 2016; Zepp et al., 2017). We leveraged a loss-of-function approach, specifically during alveologenesis, to identify distinct signaling pathways whose *in vivo* inhibition negatively impacted pAT2 self-renewal. We identified six major signaling networks, HGF, FGF, BMP, WNT, NOTCH, and IGF1. Of these, NOTCH, IGF1, and HGF are less studied. Blocking HGF had the strongest impact on organoid size. HGF is a recognized growth factor for AT2 proliferation *in vitro* (Calvi et al., 2013; Ohmichi et al., 1998). Blocking NOTCH or IGF1 also impacted clonogenicity and organoid size. The role of NOTCH in self-renewal of airway basal stem cells and differentiation is recognized (Kiyokawa and Morimoto, 2020; Tsao et al., 2016; Xu et al., 2010). Consistent with our results, blocking NOTCH signaling in AT2s is reported to cause abnormal lung development and loss of AT2s (Tsao et al., 2016). Limited information is available on the potential role of IGF1 in AT2 regulation (Ghosh et al., 2013). However, IGF1 sustains stem cell-mediated repair of tubular epithelial cells in acute kidney injury (Imberti et al., 2007). The potential *in vivo* role of these ligands in pAT2 self-renewal remains to be investigated by utilizing loss-of-function models.

Our findings regarding FGF, BMP, and WNT in organoid cultures are largely consistent with previous reports (Chung et al., 2018; Hu et al., 2020; Zepp and Morrisey, 2019). We found clonogenicity to be particularly sensitive to changes in FGF and WNT. In the adult lung, paracrine WNT signaling from a single *Wnt5a*(+) fibroblast in close proximity to adult Axin2(+) stem cells governs their behavior (Nabhan et al., 2018). C3 cells express WNT ligands including Wnt5a (data not shown). 3D reconstruction of confocal images in PN14 lungs shows that *Gli1-creERT2*-targeted GFP(+) and SFPTC(+) cells are in close spatial proximity to one another (Figure S11). Wnt2-expressing cells, which are exclusively in the C3 subcluster, are indeed juxtaposed to pAT2 cells (Figure 6). Importantly, C3 cells are also *Prcn*(+), indicating that they secrete WNT ligands (Resh, 2016) (Figure S12). In contrast, the majority of C4 cells are *Prcn*(−), raising the possibility of a paracrine WNT-mediated C3-C4 crosstalk during alveologenesis akin to that described in the adult stem cell niche (Nabhan et al., 2018).

WNT signaling and expression of its target *Axin2* have been described in AT2s during lung development and in response to injury (Basil et al., 2020). In early alveologenesis, the majority of proliferating AT2s are reported to be *Axin2*(+) (Frank et al., 2016). In our study, nearly all C4 cells are proliferating (Figure 3) and yet only 10% are *Axin2*(+) (Figure S12). This percentage remains the same in mutant C4 cells despite profound depletion of total AT2s (Figure 3). Adult lung AT2 facultative stem cells that proliferate in response to injury are also *Axin2*(+). These cells are reported to be rare, constituting between 1% and 20% of total AT2s (Nabhan et al., 2018; Zacharias et al., 2018). While we do not as yet know the significance of the rare *Axin2*(+) population in C4, they are proliferative and may represent progenitors of a unique subset of adult AT2s with proliferative potential. It is noteworthy that in the organoid cultures we found percent CFE to remain constant, regardless of the number of input pAT2s (Figure 6). This indicates that during alveologenesis only a given fraction of total pAT2s is clonogenically competent, perhaps by virtue of possessing proliferative potential. In this regard, we found a decreasing trend in the overall ratio of *Axin2*(+) pAT2s to total AT2s as pAT2s differentiated along the trajectory line. To determine whether the proliferative status of the cells is reflected in their metabolic state, we examined the expression of multiple subunits of the NADH pathway, the largest of the respiratory complexes. As shown in Figure 4D, transcripts for the NADH subunits decreased along the C4 to C10 trajectory, which largely followed the pattern of reduction in the *Axin2*(+)pAT2/total AT2 ratios shown in Figure S12. The metabolic decrease occurred strongly in C8, C9, and C10 at the inflection point, where *Sftpc*, the marker for AT2 cells, began to decrease (Figure 4A). C8, C9, and C10 also had the lowest *Axin2*(+) pAT2/total AT2 ratios (Figure S12). Metabolic transcripts were restored in C11 cells, which represent differentiated AT1 cells (Figures S5B and S4D). AT1s are thought to be *Axin2*(−), and terminally differentiated, implying loss of proliferative potential. We found residual *Axin2*(+) cells within the C11 cluster, suggesting the presence of perhaps a distinct subpopulation of AT1s that retain proliferative capacity. Developmental plasticity and reversal of the terminally differentiated state have been suggested for AT1 cells (Yang et al., 2016). Our collective observations indicate that proliferative potential may be progressively lost during differentiation of cells exiting the C4 pool. However, as each of the differentiating subclusters still retains a pool of *Axin2*(+) cells, one can speculate that the cells in early alveologenesis (PN14) may maintain residual proliferative capacity. While the validity of this concept needs to be tested, it raises potentially exciting possibilities for future treatment modalities for BPD.

Pathogenesis of BPD is the result of injury to the developing, nascent alveoli caused by the side effects of clinical interventions necessary to maintain life after preterm birth. In adult mice, H1N1 influenza causes extensive alveolar injury. In response, AT2 cells identified as *Axin2*(+);*Sftpc*(+) and referred to as AEPs undergo self-renewal and differentiate to regenerate the injured alveoli (Zacharias et al., 2018). AEPs are present in the adult human lung and account for around 29% of AT2s (Zacharias et al., 2018). Whether AEPs are present and functional in immature lungs of preterm neonates has not been discovered. We found that *AXIN2*(+);SFTPC(+) cells are in fact present in the control human neonatal lungs and account for nearly 28% of the total pAT2 cell population, a remarkably conserved parallel to the mature adult human lungs (Figure 7). Intriguingly, in BPD lungs this ratio (and not the absolute number) is increased to 36.5%, likely caused by an incomplete or blunted response to injury. This increase is also observed in the mouse DKO lungs in which the overall ratio of *Axin2*(+) pAT2s to total pAT2s is nearly twice as large in the mutant lungs compared with controls (20% and 10.2%, respectively) (Figure S12). However, while the relative pool of “regenerative” cells is larger, the relative number of proliferating pAT2s is smaller in BPD lungs compared with controls, again similar to the mouse DKO lungs. Reduced *PDGFRa* is a primary feature of BPD (Popova et al., 2014) and may indeed be a potential cause of failed mesenchymal-pAT2 crosstalk, ultimately accounting for lack of pAT2 responsiveness in BPD. In addition, altered expression of several of the mouse ligands identified here have been found in human BPD (Alejandre-Alcazar et al., 2007; Bourbon et al., 2005; Chao et al., 2020; Lassus et al., 2003; Lecarpentier et al., 2019; Seedorf et al., 2016; Sucre et al., 2016). Collectively these data indicate that BPD may more likely be caused by failure in recruitment rather than lack of presumptive regenerative epithelial cells. The latter has obvious implications for the pathogenesis of BPD and may offer novel preventive or therapeutic strategies for this neonatal chronic lung disease.

### Limitations of the study

The present study was in large part focused on genetic analysis of a compound mutation that affects TGFβ signaling in a select population of mesenchymal cells. In addition, the analyses centered around scRNA-seq and limited analysis of the protein products of the genes involved. While highly focused, such analyses may fall short of revealing the complex interactions that constitute cellular and physiologic function in the mutant lungs.

## STAR★METHODS

### RESOURCE AVAILABILITY

#### Lead contact

Further information and requests for the resources and reagents should be directed to the lead contact, Parviz Minoo (minoo@usc.edu).

#### Materials availability

Mouse line generated in this study are available to distribute upon approval of the institutional MTA. The raw and analyzed scRNAseq data in this study are deposited in GEO database: GSE172059.

#### Data and code availability

The raw and analyzed scRNAseq data in this study are deposited in GEO database: GSE172059.

This paper does not report original code.

Any additional information required to reanalyze the data reported in this paper is available from the lead contact upon request.

### EXPERIMENTAL MODEL AND SUBJECT DETAILS

#### Mouse breeding and genotyping

All animal studies were conducted strictly according to protocols approved by the USC Institutional Animal Care and Use Committee (IACUC) (Los Angeles, CA, USA). The mice were housed and maintained in pathogen-free conditions at constant room temperature (20–22°C), with a 12 h light/dark cycle, and free access to water and food. *Gli1-cre*^*ERT2*^*, Rosa26*^*mTmG*^*, CAG*^*Tomato*^*, Alk5*^*(fox/flox)*^ and *Tbr2*^*(flox/flox)*^ mice were purchased from the Jackson Laboratory. *CAG*^*Tomato*^ mice express Tomato Red in presence of activated Cre (Stock No: 007909). *Rosa26*^*mTmg*^ express GFP in presence of activate Cre (Stock No: 007676). *Sftpc-GFP* mice express GFP in cells that express the surfactant protein gene C, mostly progenitors of AT2 and differentiated AT2 cells. *Gli1-cre*^*ERT2*^*;Rosa26*^*mTmG*^ *mice* (control) were generated by breeding *Gli1-cre*^*ERT2*^ and *Rosa26*^*mTmG*^ mice. *Gli1-cre*^*ERT2*^*;Rosa26*^*mTmG*^*; Alk5*^*(fox/flox)*^*; Tbr2*^*(flox/flox)*^ (abbreviated as DKO) mice were generated by breeding control mice with the *Alk5*^*(fox/flox)*^ and *Tbr2*^*(flox/flox)*^ mice. *Gli1-cre*^*ERT2*^*; CAG*^*Tomato*^ mice were generated by breeding *Gli1-cre*^*ERT2*^ and CAG^Tomato^ mice. *Gli1-cre*^*ERT2*^*;Sftpc-GFP; Alk5*^*(fox/flox)*^*; Tbr2*^*(flox/flox*^ mice were generated by breeding *Gli1-cre*^*ERT2*^*; Alk5*^*(fox/flox)*^*; Tbr2*^*(flox/flox)*^; with *Sftpc-GFP* mice.

Genotyping of the transgenic mice was performed by PCR with genomic DNA isolated from mouse tails. The primers for mouse genotyping are listed in Table S1.

#### Mouse lung tissue

Mice were euthanized by CO_2_ inhalation at the time of tissue harvest. Sex of the mice was not noted. Chest cavity was exposed and lungs cleared of blood by perfusion with cold PBS via the right ventricle. Lungs were inflated with 4% formaldehyde under constant pressure of 30 cm water and allowed to fix overnight in refrigerator. Tissue was dehydrated through a series of ethanol washes after which they were embedded in paraffin and sectioned.

#### 3D alveolar organoid culture

3D alveolar organoid assays were performed as originally described (Barkauskas et al., 2013) with some modifications. Briefly, *Gli1Cre*^*ERT2*^*;CAG*^*Tomato*^ pups were given tamoxifen at PN2 and the lungs were isolated at PN14. Lungs were also isolated from *Sftpc-GFP* pups at PN14. Single cell suspensions were made from both groups of mouse lungs as described above. GFP labelled AT2 cells and Tomato labelled mesenchymal cells were sorted out by FACs. Previous studies indicated a 1:10 mix of AT2 and mesenchymal cells as the condition best optimized for organoid’s growth. With this ratio as baseline, a combination of different number of above sorted AT2 and mesenchymal cells, were mixed in 50% Matrigel (growth factor reduced, phenol-red free) (Corning), gelled on Costar Transwell Polyester Membrane Inserts (Fisher Scientific), and cultured in air-liquid interface in basal medium (DMEM/F12, 10%FBS, 1x insulin/transferrin, 1xPSA). Fresh media was replaced every two days, and the organoid growth was monitored and documented under the inverted fluorescent microscope periodically. Ligand-receptor interactions discovered between AT2 and mesenchymal cells were functionally examined by inhibiting the indicated pathways. Inhibitors were carefully selected from literature based on their specificity, quality and effect. A search of the literature was used to determine the range of the concentration to be used for each inhibitor. A gradient of different concentrations around the range were tested, and the lowest concentration with phenotype distinct from the control was the final concentration used for the treatment in the study. Please see the Table S1 for the list of inhibitors and the final concentration used. Inhibitors were added at the time of the first media change, and new inhibitors were added upon each media change or every other day.

#### Human fetal lung

BPD and non-BPD postnatal human lung tissues were provided by the International Institute for the Advancement of Medicine and the National Disease Research Interchange, and were classified exempt from human subject regulations per the University of Rochester Research Subjects Review Board protocol (RSRB00056775). The control lung samples were from female and male neonates born at term gestation (40 weeks) who died of non-pulmonary causes. The BPD samples were from female and male neonates born prematurely at between 23 weeks and 27 weeks of gestation and died with a diagnosis of BPD.

### METHOD DETAILS

#### Tamoxifen administration

A single dose of Tamoxifen (8mg/mL in peanut oil) was administered by oral gavage to neonates at postnatal day 2 (P2, 400 μg each pup) with a plastic feeding needle (Instech Laboratories, PA). Neonatal lungs were collected between P7 and P30 for morphological, immunohistochemical, cellular and molecular biological analyses.

#### Immunohistochemistry

H&E staining was performed as usual, and morphometric measurements were made using ImageJ. Immunofluorescent staining was performed as previously described using paraffin-embedded lung sections (Li et al., 2019). In brief, five micrometer (μm) tissue sections were deparaffinized, rehydrated and subjected to antigen retrieval. After blocking with normal serum, the sections were probed with primary antibodies at 4°C overnight. Combinations of fluorescein anti-mouse and Cy3 anti-rabbit or anti-goat IgG secondary antibodies (Jackson ImmunoResearch Laboratories, ING) were applied to detect specific primary antibodies. Nuclei were counterstained with 4′,6-diamidino-2-phenylindole (DAPI). Primary antibodies used and their sources are listed in the Key Resources Table. Images were made with Leica DMi8 fluorescence microscope and processed with Leica LAS X and ImageJ. MLI or mean linear intercept was determined by measurement of the mean interalveolar septal wall distance determined by the number of interruptions that four drawn lines make in each field. Five to six images (10× magnification) from each lung were used to manually count the intercepts, and at least five random fields were examined per tissue.

#### RNAScope

Samples were fixed in either 10% neutral buffered formalin, dehydrated with ethanol and embedded in paraffin wax or fixed in 4% paraformaldehyde and embedded in OCT compound. Sections from paraffin (5 μm) and OCT (20 μm) blocks were processed using standard pretreatment conditions for each per the RNAscope multiplex fluorescent reagent kit version 2 (Advanced Cell Diagnostics) assay protocol. TSA-plus fluorescein, Cy3 and Cy5 fluorophores were used at different dilution optimized for each probe. RNAScope was followed, when needed, by IF staining same as described above in immunohistochemistry. RNAScope probes used are listed in the KRT Table. Micrographs were acquired with a laser scanning confocal fluorescence microscope (Zeiss LSM780) and processed with ImageJ and Imaris.

#### Mouse lung single-cell dissociation

Single-cell suspension was prepared as described in Adam et al. (2017) with all the procedures run on ice or in cold room. Mice were euthanized and the lungs were perfused with PBS as described above. The lungs were inflated with cold active protease solution (5 mM CaCl2, 10 mg/mL Bacillus Licheniformis protease), dissected and transferred to a petri dish, and the heart, thymus and trachea were removed. The lobes were minced using a razor blade. The minced tissue was then immersed in extra cold active protease solution for 10 min and triturated using a 1 mL pipet. This Homogenate was transferred to a Miltenyi C-tube with 5mL HBSS/DNase (Hank’s Balanced Salt Buffer) and the Miltenyi gentleMACS lung program was run twice. Subsequently, this suspension was passed through a 100μm strainer, pelleted at 300 g for 6 minutes, suspended in 2mL RBC (Red Blood Cell) Lysis Buffer (BioLegend) and incubated for 2 min. At this point, 8mL HBSS was added and centrifuged again. The pellet was suspended in HBSS, filtered through a 30um strainer and pelleted and suspended in MACS separation buffer (Miltenyi Biotec) with 2% FBS for FACs and in HBSS/0.01% BSA for 10x single cell sequencing. Cell separation and viability were confirmed under the microscope and through Vi-CELL Cell Counter after staining with Trypan blue.

#### Flow cytometry and cell sorting

Single cell suspensions were gated on viability by staining with ghost dye red 780, then gated on singlets and separated on GFP from *Gli1-cre*^*ERT2*^*; Rosa26*^*mTmG*^ or *Sftpc-GFP* labeling or Tomato from *Gli1-cre*^*ERT2*^*; CAG*^*Tomato*^ labeling. Cell sorting was performed on a BD FACS Aria II at stem cell Flow Cytometry Core at Keck school of medicine USC. For cell counting, cell number and percentage as of its appropriate group were recorded and used to calculate the difference between control and mutant. For Organoid culture, cells were sorted in DMEM containing 10% FBS. For RNA, cells of interest were collected in Trizol-LS reagent (ThermoFisher). The RNA was isolated according to the manufacture’s protocol. Following RNA purification, cDNA was generated using the SuperScript IV First-Strand Synthesis System (ThermoFisher). Quantitative Real-Time PCR was then performed on a Light Cycler (Roche) or 7900HT fast real-time PCR system (Applied Biosystems) using SYBR green reagents (ThermoFisher).

#### Single-cell RNA-sequencing

We used the Chromium Single Cell 3′ kit (v3) of 10x Genomics for performing scRNA-sequencing. The single cell suspension prepared above was loaded onto a Chromium Single-Cell instrument (10x Genomics) to generate single-cell barcoded droplets (GEMs) as detailed in manufacture’s protocol. The resulting libraries were pooled and sequenced across two lanes on an Illumina HiSeq2500 instrument in High-output mode by Novogene. Reads were aligned and subsequent analyses performed using the Cell Ranger (Pipeline). We obtained 68k reads per cell with a median genes per cell of 1,017 and median UMI count per cell of 2,040. The accession number for the scRNA-seq data is GEO database: GSE172059.

#### Bioinformatics

The 10x Genomics’ Cell Ranger pipeline was used to demultiplex raw base call (BCL) files into FASTQ files, perform alignment, filtering, barcode counting, and UMI counting, combine and normalize counts from multiple samples, generate feature-barcode matrices, run the dimensionality reduction, clustering, and gene expression analysis. Quality control was done additionally in Partek Flow to filter out cells with excess mitochondrial reads and possible doublets and remove batch effects. Loupe Browser was used for data visualization and analysis including identification of differentially expressed genes and marker genes, cell type classification, cell re-clustering, cell counting, gene expression comparative analysis between different cell clusters at both cell and population levels. DE genes derived above were imported into IPA (Ingenuity Pathway Analysis from QIAGEN) for pathway and functional analysis. To construct single cell pseudo-time trajectory and to identify genes that change as the cells undergo transition, Monocle 3 algorithm was applied to the cells from clusters 4 to 11. Interactions between cell types were predicted using a published Fantom5 Cell Connectome dataset linking ligands to their receptors (STAR Methods) (Ramilowski et al., 2015).

### QUANTIFICATION AND STATISTICAL ANALYSIS

Statistical analysis was performed in JMP pro 15. A two-tailed non-parametric Mann-Whitney U-test was used for the comparison between two experimental groups and a one-way ANOVA was used for multiple comparisons. Data were considered significant if *p* < *0.05*.

## Supplementary Material

1

2

## Figures and Tables

**KEY RESOURCES TABLE:** 

REAGENT or RESOURCE	SOURCE	IDENTIFIER

Antibodies		

GFP	Santa Cruz	Cat#: SC-9996; RRID:AB_627695
SFTPC	EMD Millipore Corp.	Cat #: AB3786; RRID:AB_91588
HOPX	Santa Cruz	Cat #: SC-30216; RRID:AB_2120833
MKI67	R&D System	Cat #: AF7649; RRID:AB_2687500
PDGFRA	Cell Signaling	Cat #: 3174
ACTA2	Abcam	Cat#: AB5694; RRID:AB_2223021
AQP5	Alomone	Cat #: AQP-005; RRID:AB_2039736
SFTPC (Human Tissue)	LSBio	Cat #: LS-B10952
KI67 (Human Tissue)	Cell Signaling	Cat #: 9449T; RRID:AB_2715512

Biological samples		

Human fetal lungs	The International Institute for the Advancement of Medicine and the National Disease Research Interchange	https://ndriresource.org/

Chemicals, peptides, and recombinant proteins		

Tamoxifen	Sigma	Catalog No. T5648
Capmatinib	Selleckchem	Catalog No. S2788
Grem2	R&D Systems	Catalog No. 2069-PR-050
DAPT	Selleckchem	Catalog No. S2215
BGJ398	Selleckchem	Catalog No. S2183
XAV939	Selleckchem	Catalog No. S1180
PPP	Selleckchem	Catalog No. S7668

Critical commercial assays		

Chromium Single Cell 3′ Library & Gel Bead Kit v3	10x Genomics	Catalog No. 1000092
Single Cell In-Drop RNA-sequencing	Novogene Corporation	GEO database: GSE172059
RNAscope® Multiplex Fluorescent Reagent Kit V2	Advanced Cell Diagnostics	Catalog No. 323100

Deposited data		

Raw and analyzed scRNAseq data	This paper	GEO database: GSE172059
Inhibitor concentrations tested and used in organoid culture	This paper	Table S1

Experimental models: Organisms/strains		

*Gli1-cre^ERT2^*	Jackson Laboratories	Catalog No. 007913
*Rosa26^mTmG^*	Jackson Laboratories	Catalog No. 007676
*CAG^Tomato^*	Jackson Laboratories	Catalog No. 007914
*Alk5 ^flox/flox^*	Jackson Laboratories	Catalog No. 028701
*Tbr2 ^flox/flox^*	Jackson Laboratories	Catalog No. 012603

Oligonucleotides		

*Sftpc-RNAscope probe*	Advanced Cell Diagnostics	Catalog No. 314101-C1
*Hopx-RNAscope probe*	Advanced Cell Diagnostics	Catalog No. 405161-C3
*Aqp5-RNAscope probe*	Advanced Cell Diagnostics	Catalog No. 430021-C2
*Fgfr2-RNAscope probe*	Advanced Cell Diagnostics	Catalog No. 316851-C1
*Pdgfra-RNAscope probe*	Advanced Cell Diagnostics	Catalog No. 480661-C2
*Wnt2-RNAscope probe*	Advanced Cell Diagnostics	Catalog No. 313601-C1
*Fgf10-RNAscope probe*	Advanced Cell Diagnostics	Catalog No. 446371-C1
*Fgf2-RNAscope probe*	Advanced Cell Diagnostics	Catalog No. 443501-C2
*Mfap4-RNAscope probe*	Advanced Cell Diagnostics	Catalog No. 421391-C1
*MKI67-RNAscope probe*	Advanced Cell Diagnostics	Catalog No. 416771-C3
*AXIN2-RNAscope probe*	Advanced Cell Diagnostics	Catalog No. 400241-C2
RNAscope® 3-plex Positive Control Probe_Mm	Advanced Cell Diagnostics	Catalog No. 320881
RNAscope® 3-plex Negative Control Probe	Advanced Cell Diagnostics	Catalog No. 320871
Primers for mouse strain genotyping	This paper	Table S1
Primers for quantitative RT-PCR	This paper	Table S1

Software and algorithms		

Cell Ranger R kit	10x Genomics	https://support.10xgenomics.com/single-cell-gene-expression/software/pipelines/latest/rkit
Loupe Cell Browser	10x Genomics	https://support.10xgenomics.com/single-cell-gene-expression/software/downloads/latest#loupe
Partek Flow 9.0.20.1104	Partek Inc	https://www.partek.com/partek-flow/
Monocle 3	Trapnell Lab	https://cole-trapnell-lab.github.io/monocle3/
IPA	Qiagen	https://digitalinsights.qiagen.com/products-overview/discovery-insights-portfolio/analysis-and-visualization/qiagen-ipa/
Image J	NIH	https://imagej.nih.gov/ij/
STAR 2.5	PMCID: PMC3530905	https://github.com/alexdobin/STAR
JMP pro 15	Statistical Discovery	https://www.jmp.com/en_us/software/predictive-analytics-software.html
Fantom5 Cell Connectome	FANTOM5 project	https://fantom.gsc.riken.jp/5/suppl/Ramilowski_et_al_2015/vis/#/hive
Imaris	BitPlane	http://www.bitplane.com/imaris/imaris
R 3.2	R Project	https://www.r-project.org/
LAS X	Leica	https://www.leica-microsystems.com/products/microscope-software/p/leica-las-x-ls/

## Highlights

- Lung alveolar type 2 progenitors (pAT2s) are heterogeneous during alveologenesis
- Blocking TGFβ in SCMF fibroblasts reduces their number and blocks alveologenesis
- SCMF signaling regulates proliferation of unique pAT2 population in alveologenesis
- An evolutionarily conserved mechanism appears to govern pAT2s in neonatal human lungs
