# Supplementary material for: Hedgehog-responsive PDGFRa(+) fibroblasts maintain a unique pool of alveolar epithelial progenitor cells during alveologenesis
Source: Cell Rep. Author manuscript; Available in PMC 2022 Jun 15. (PMC9199394; doi:10.1016/j.celrep.2022.110608)
Supplement: 1 [file NIHMS1795985-supplement-1.pdf]

Cell Reports, Volume *39*

Supplemental information

Hedgehog-responsive PDGFRa(+) fibroblasts maintain a unique pool of alveolar epithelial progenitor cells during alveologenesis

Feng Gao, Changgong Li, Soula Danopoulos, Denise Al Alam, Neil Peinado, Sha Webster, Zea Borok, GoleNaz Adeli Kohbodi, Saverio Bellusci, and Parviz Minoo

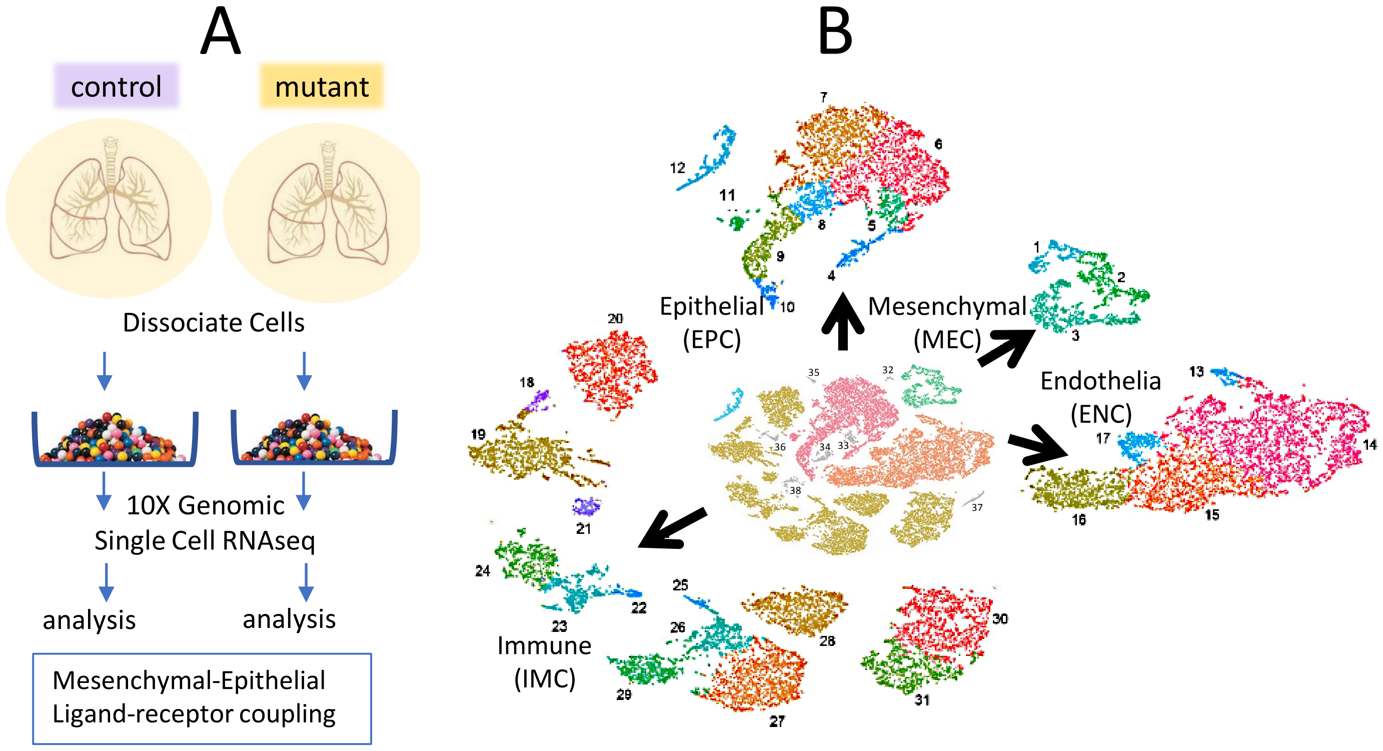

C

		Ctrl	Mut	%PS	Type
Mesen	1	75	135	27.71	Mesen-1
	2	260	345	13.13	Mesen-2
	3	689	71	-81.63	Mesen-3
Epi	4	283	5	-96.59	AT2-1
	5	225	45	-67.18	AT2-2
	6	1342	528	-44.28	AT2-3
	7	395	979	41.74	AT2-4
	8	205	149	-16.73	AT2-5
	9	435	262	-25.69	AT2-6
	10	125	86	-19.38	AT2-7
	11	112	46	-42.54	AT1
	12	106	301	47.19	Airway
Endo	13	117	6	-90.42	Endo-1
	14	1511	1872	9.75	Endo-2
	15	589	950	22.57	Endo-3
	16	582	458	-12.84	Endo-4
	17	176	160	-5.69	Endo-5
Immune	18	86	69	-11.89	Myeloid-1
	19	816	601	-16.08	Myeloid-2
	20	856	645	-14.97	Myeloid-3
	21	57	137	40.46	Myeloid-4
	22	98	19	-68.03	Monocyte-1
	23	359	126	-48.76	Monocyte-2
	24	359	283	-12.76	Monocyte-3
	25	65	42	-22.38	Lymphocyte-1
	26	308	182	-26.58	Lymphocyte-2
	27	673	821	8.98	Lymphocyte-3
	28	424	706	24.08	Lymphocyte-4
	29	352	276	-13.02	Lymphocyte-5
	30	429	1077	42.26	Neutrophils-1
	31	18	839	95.72	Neutrophils-2
Doublet	32	44	10	-63.52	Epi+Mesen
	33	92	66	-17.36	AT2+Endo
	34	89	120	13.92	AT2+Club
	35	11	54	65.63	AT2+Immune
	36	96	48	-34.16	Epi+Immune
	37	20	124	71.77	Endo+Immune
	38	104	88	-9.26	Mono+Lymphocyte

Supplemental Figure 1: Molecular Phenotype of PN14 Lungs at Single Cell Resolution, Related to Figures 2 and 3. (A) Schematic of the approach used for single cell RNAseq. (B) tSNE representation of the combined single cell sequencing data from Tamoxifen-treated *Gli1-creERT2;Rosa26mTmg* (control and *Gli1-creERT2; Alk5(flox/flox);TbR2(flox/flox), (*DKO) control lungs using an iterative unbiased clustering strategy reveals a diversity of cell types at PN14 (n=26,401). The 4 major cell compartments are further re-clustered into more defined sub-clusters that are numbered, in different colors and shown by arrows. (C) Table showing the identity of, the total number of cells in, and percent alterations between the 38 sub-clusters identified in the control versus mutant PN14 lungs (%PS or population shift). Sub-clusters 32 to 38 are doublets.

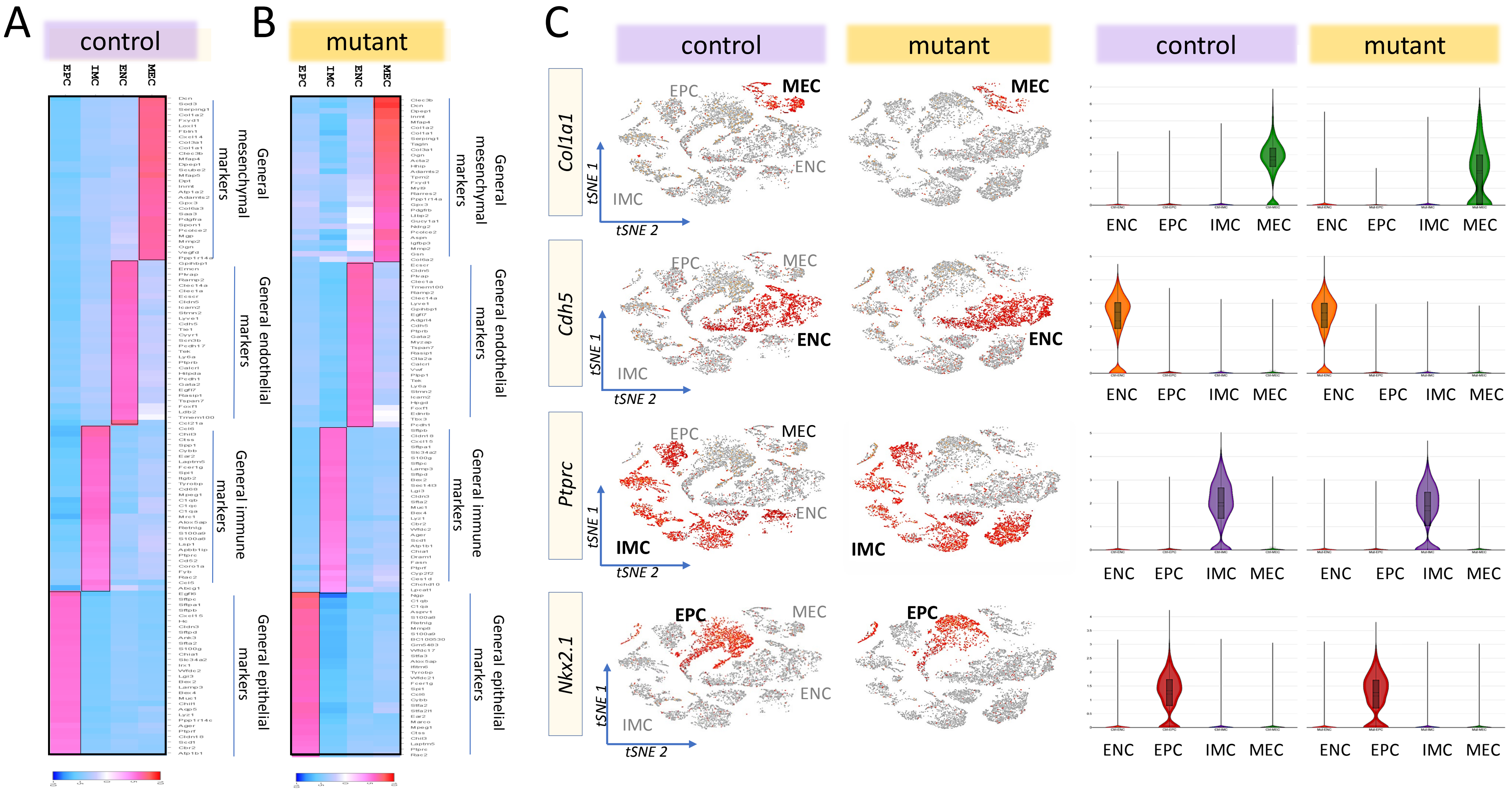

Supplemental Figure 2: Diversity of Distal Lung Cell Types at PN14 in Control and Mutant Lungs, Related to Figures 2 and 3. (A and B) Heat maps of cell type enriched genes in each of the four major clusters comprising the scRNAseq data from control and mutant PN14 total lungs. The four major clusters, Mesenchymal (MECs), Epithelial (EPCs), Endothelial (ENCs) and Immune (IMCs). Cells were identified by analysis of cell type-enriched transcripts in each of these clusters. (C) tSNE representation of select enriched transcripts for MECs (Col1a1), EPCs (Nkx2.1), ENCs (Cdh5) and IMCs (Ptprc) in control and mutant lungs. (D) Violin plot representation of the abundance of transcripts shown in (C).

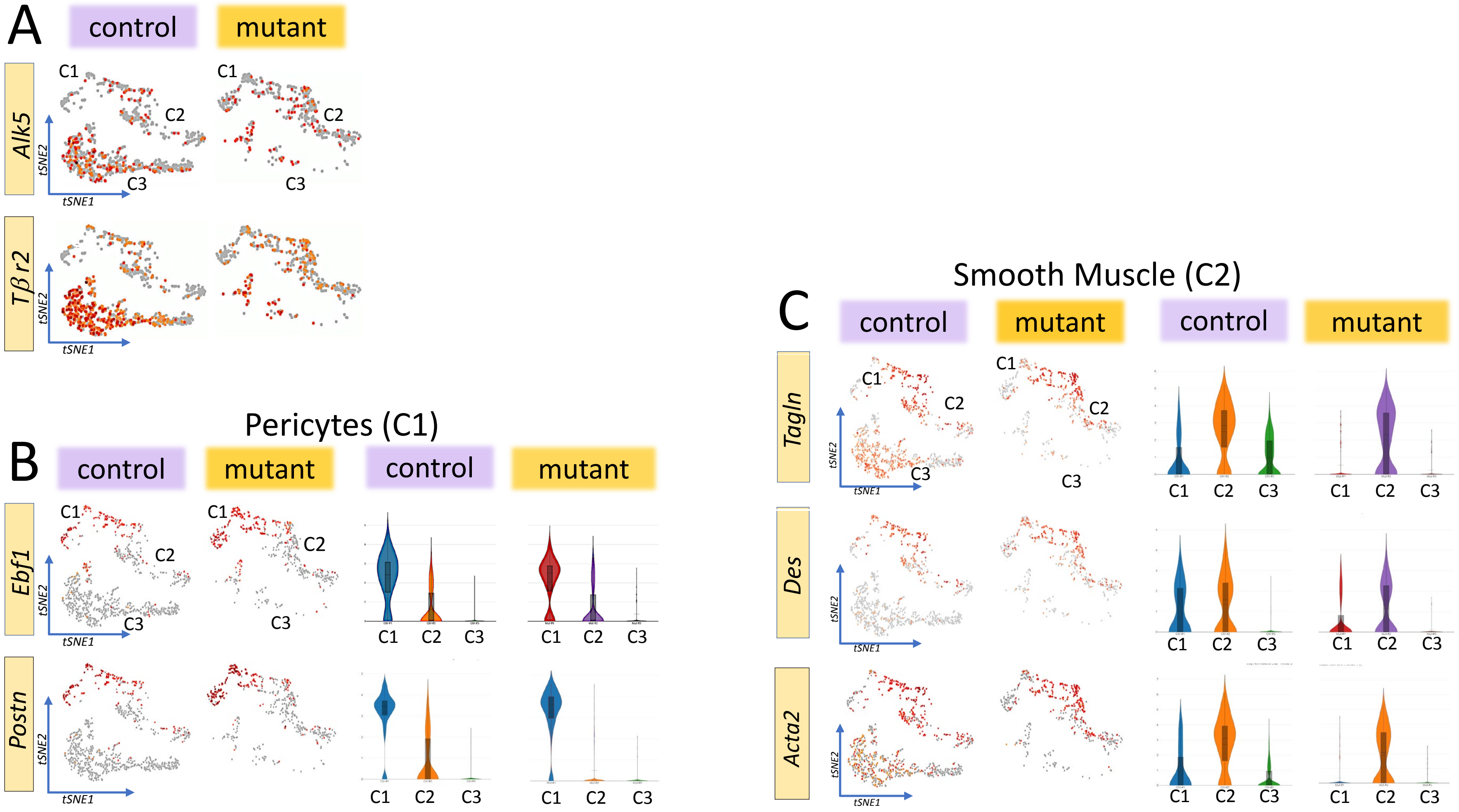

Supplemental Figure 3: Defining Distinct Mesenchymal Sub-clusters C1 and C2 in PN14 Lungs, Related to Figure 2. (A)) tSNE representation of the scRNAseq data for the two cognate TGFb receptors, *Alk5* (type I receptor) and *Tbr2* (type II receptor) in control and mutant mesenchymal sub-clusters C1, C2 and C3. The C3 mesenchymal sub-cluster includes the highest concentration of cells expressing both *Tbr2* and *Alk5.* (B) tSNE representation and violin plot showing quantification of enriched Postn and Ebf1 transcripts, two recognized markers of Pericytes in C1. (C) Similar analysis for enriched *Acta2*, *Des*, and *Tagln* transcripts that are well-established markers of smooth muscle myofibroblasts in C2.

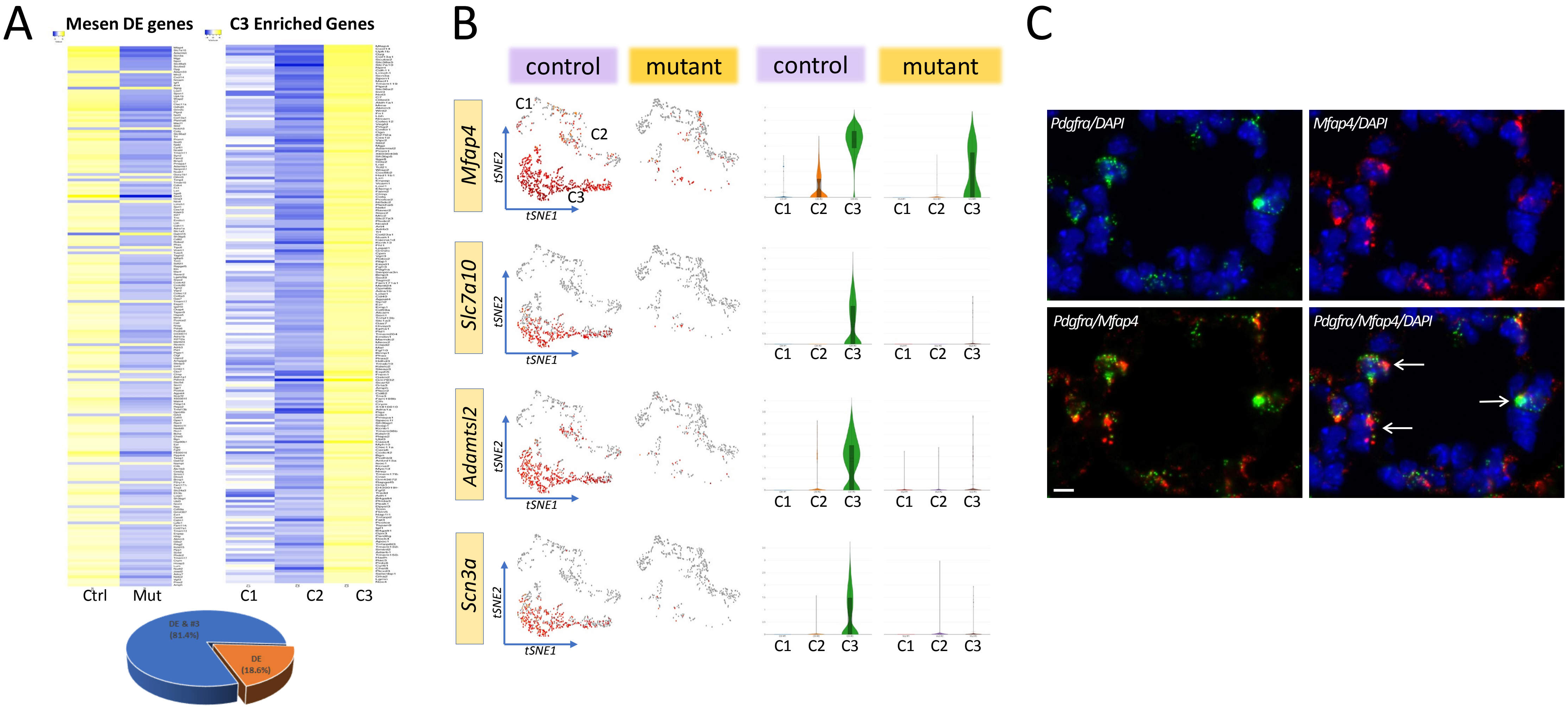

Supplemental Figrue 4: 80% of Differentially Expressed Transcripts between Control and Mutant MECs (C1+C2+C3) are Enriched in Control C3 and Lost in the Mutant, Related to Figures 2 and 5. (A) Heat map of differentially expressed (DE) mesenchymal transcripts between control (Ctrl) and mutant (Mut) lungs compared to C3 enriched genes amongst the mesenchymal sub-clusters in the control lung. (B) tSNE representation of select C3 enriched transcripts, *Mfap4, Slc7a10, Adamtsl2* and *Scn3a* and violin plot representation of their relative abundance in control versus mutant lungs. (C) RNAscope localization of *Mfap4* with *Pdgfra*(+) cells. Arrows point at double positive cells. Scale bar: 10um.

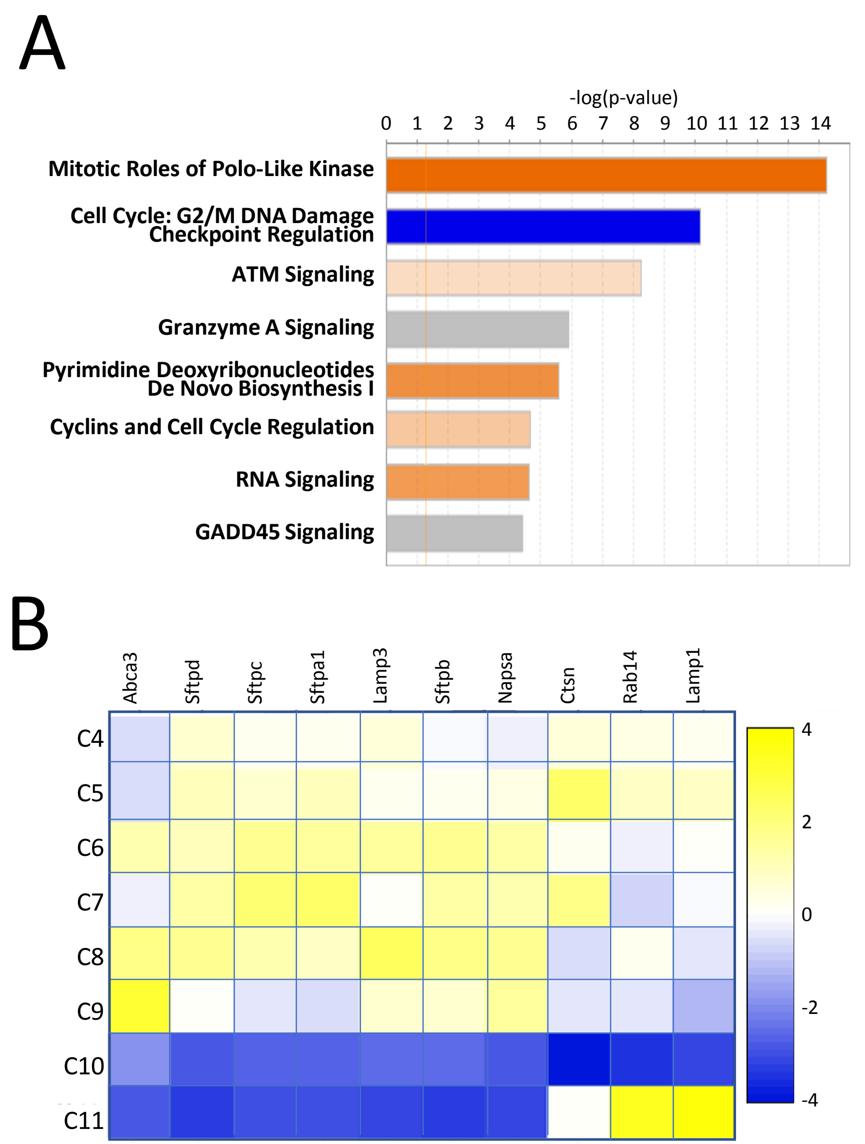

Supplemental Figure 5: Defining Distinct Epithelial Sub-Clusters in PN14 EPCs, Related to Figures 3 and 4. (A) Pathway analysis for C4-enriched transcripts. (B) Heatmap of maturing pAT2 signature genes in epithelial subclusters C4 to C11.

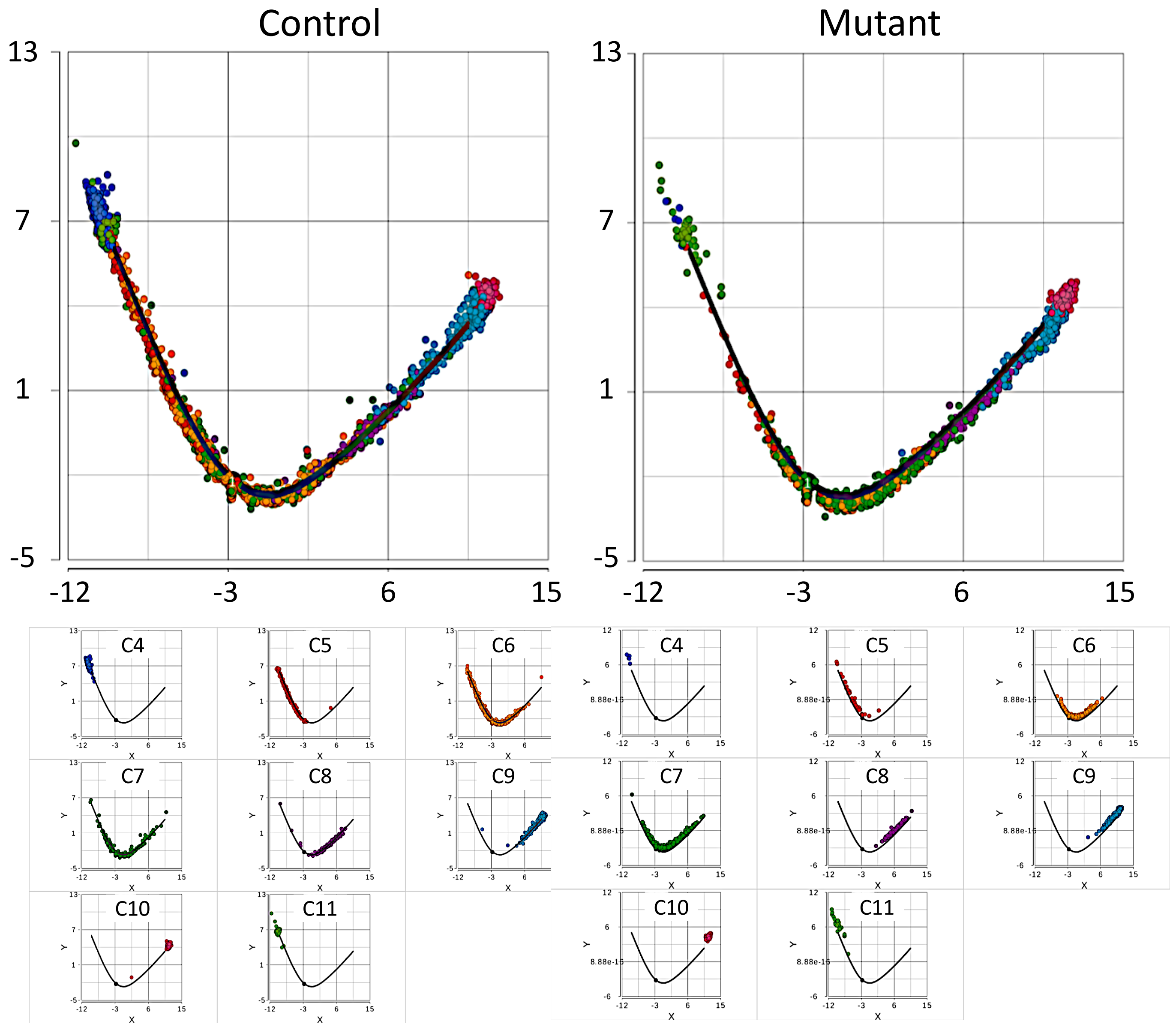

Supplemental Figure 6: Defining Distinct Epithelial Sub-Clusters in PN14 EPCs, Related to Figures 3 and 4. Pseudo-time trajectory analysis of cells showing predicted AT1, AT1/AT2, and AT2 differentiation lineage relationship model among epithelial cell subclusters C4 to C11 in control and mutant lungs.

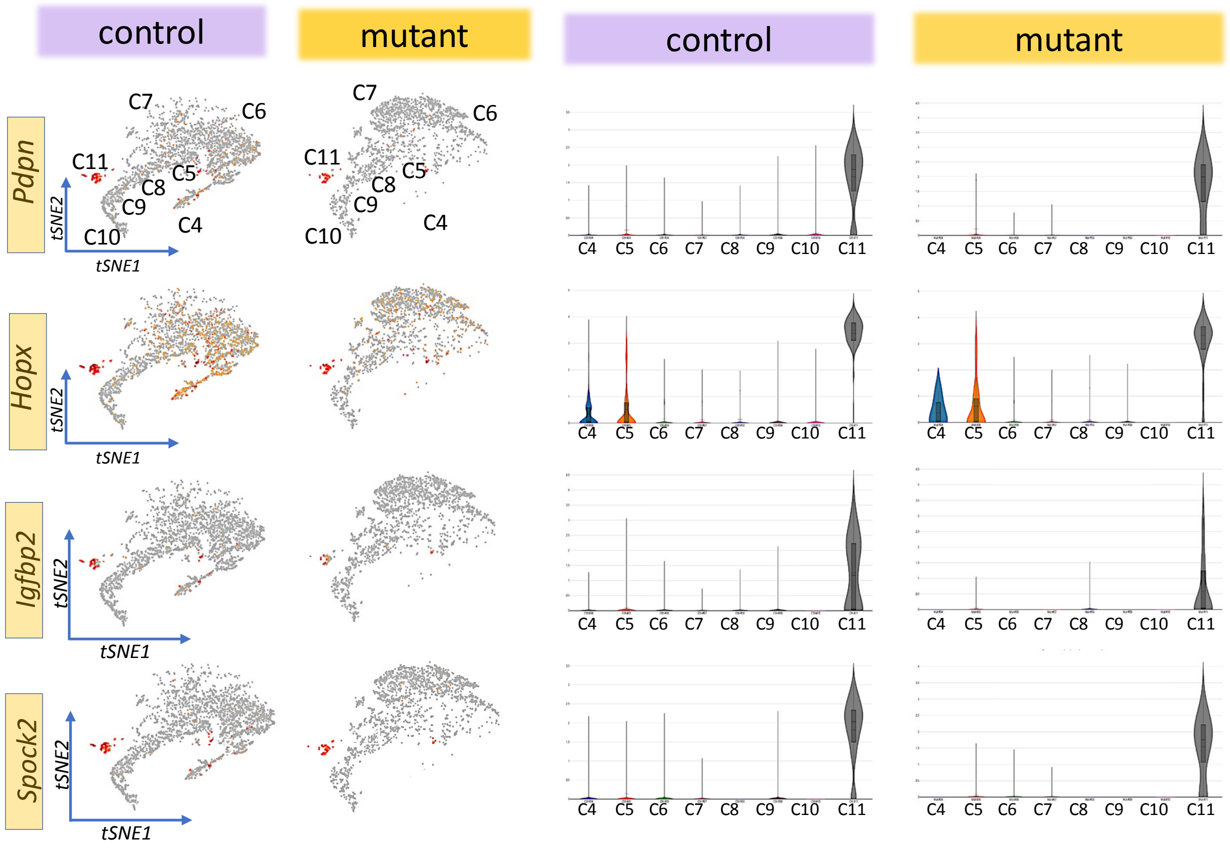

Supplemental Figrue 7: C11 Consists of Mature AT1 Cells, Related to Figures 3 and 4. tSNE representation of four genes , *Pdpn, Hopx, Igfbp2* and *Spock2*, known to be expressed in mature AT1 cells and violin representation of their expression level in C11 sub-cluster.

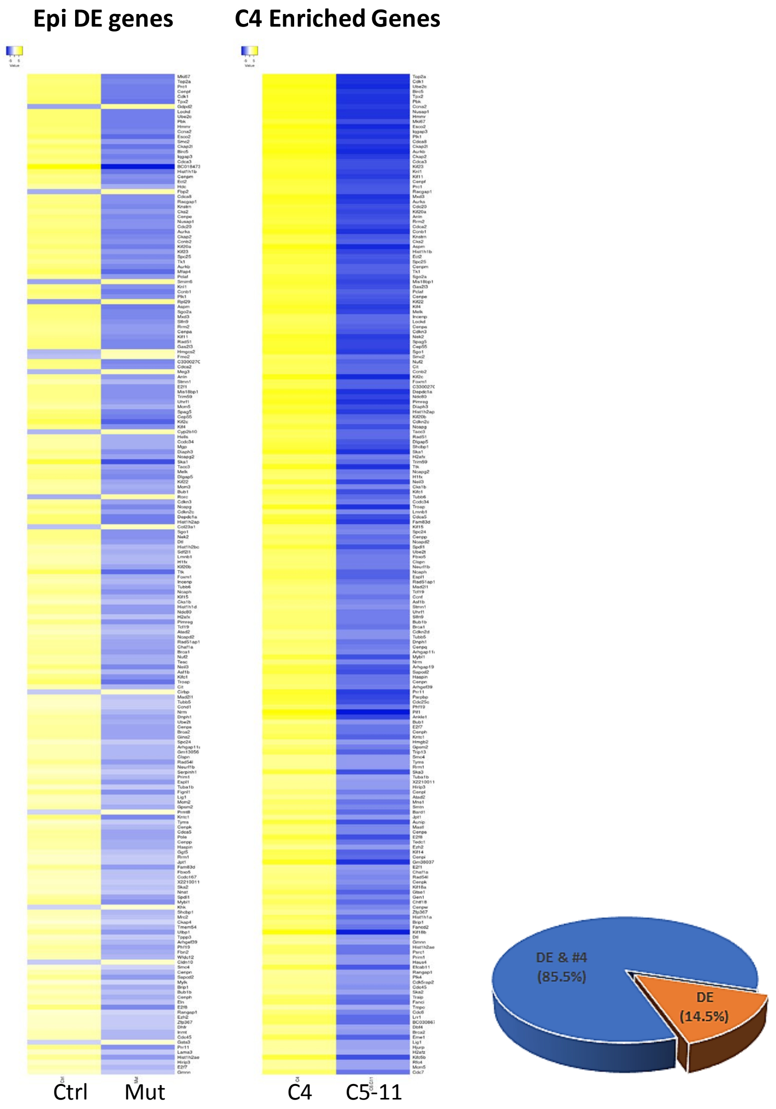

Supplemental Figrue 8: 85% of the DE Genes in the Mutant Lung Originated from C4, , Related to Figures 3, 4 and 5. Heat map of differentially expressed (DE) epithelial transcripts between control (Ctrl) and mutant (Mut) lungs compared to C4 enriched genes versus the combined enriched transcripts in C5 to C11 sub-clusters. Pie graph representation of the relative percent transcripts enriched and DE in C4.

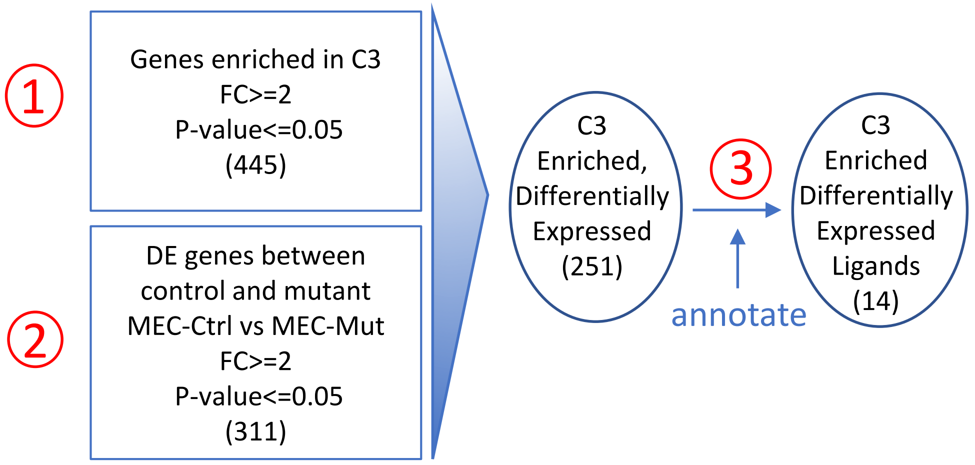

Gene	FC (Enrichment)	P-value (Enrichment)	FC (DE)	P-value (DE)
Bgn	2.42	2.18E-06	-2.32	6.70E-04
Bmp3	3.32	6.01E-09	-4.19	1.86E-07
Bmp4	2.31	9.31E-03	-2.32	7.68E-04
Col6a3	2.34	1.88E-02	-2.77	4.79E-05
Cp	2.29	3.56E-05	-3.07	4.46E-02
Cyr61	2.47	2.58E-05	-4.52	2.72E-08
Fgf10	4.19	1.69E-07	-2.72	1.44E-02
Fgf2	2.99	5.57E-06	-3.04	7.68E-04
Fn1	4.19	1.17E-14	-3.23	1.95E-06
Hgf	2.61	1.63E-02	-3.04	3.49E-02
Igf1	2.43	1.42E-05	-5.98	1.05E-11
Mfap5	2.23	5.96E-05	-2.15	4.90E-03
Npnt	5.55	1.74E-20	-7.32	3.01E-16
Wnt2	7.22	4.77E-15	-2.37	1.92E-02

Panel A

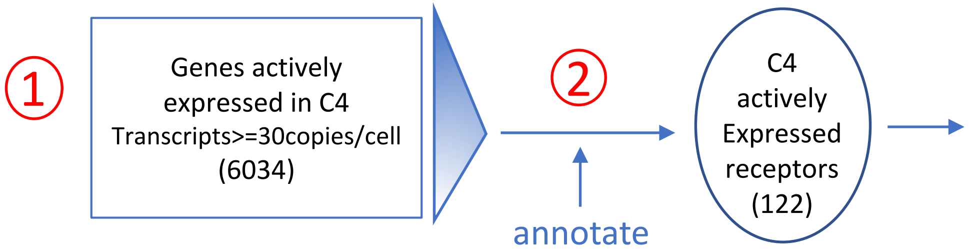

Gene	Median Normalized Average	Copies/Cell
Bmpr1a	0.41	86.81
Bmpr2	0.50	105.95
Fgfr2	0.66	139.79
Fzd5	0.15	31.61
Igf1r	0.15	31.61
Lrp6	0.43	89.93
Met	0.46	96.6
Notch1	0.37	77.46

Panel B

Supplemental Figrue 9: Ligands & Receptors Used in L-R Pairing, Related to Figure 5. Panel (A): We identified ligands enriched in C3 and differentially expressed between control and mutant lungs by using the computational algorithm shown. Initial screening identified 445 genes enriched in C3 (step 1). We next identified 311 transcripts differentially expressed (DE) in the total mesenchymal population between control and mutant lungs (Step2). 251 overlapping transcripts were identified between outcomes of Step 1 and Step 2. Of the 251 common transcripts, 14 ligands were identified by annotation (Step 3). Ligands related to ECM-based signaling are in RED font. Panel (B): Potential epithelial receptors were identified by first selecting actively expressed genes whose transcript representation in the scRNA data of C4 sub-cluster was equal or greater than 30 copies per cell (step1). This computation yielded a total of 6034 genes. Amongst these we identified 122 annotated actively expressed receptor genes (step2). Ligand-Receptor pairing using the STAR method identified 8 receptors that matched the ligands constituting the 6 principal signaling pathways.

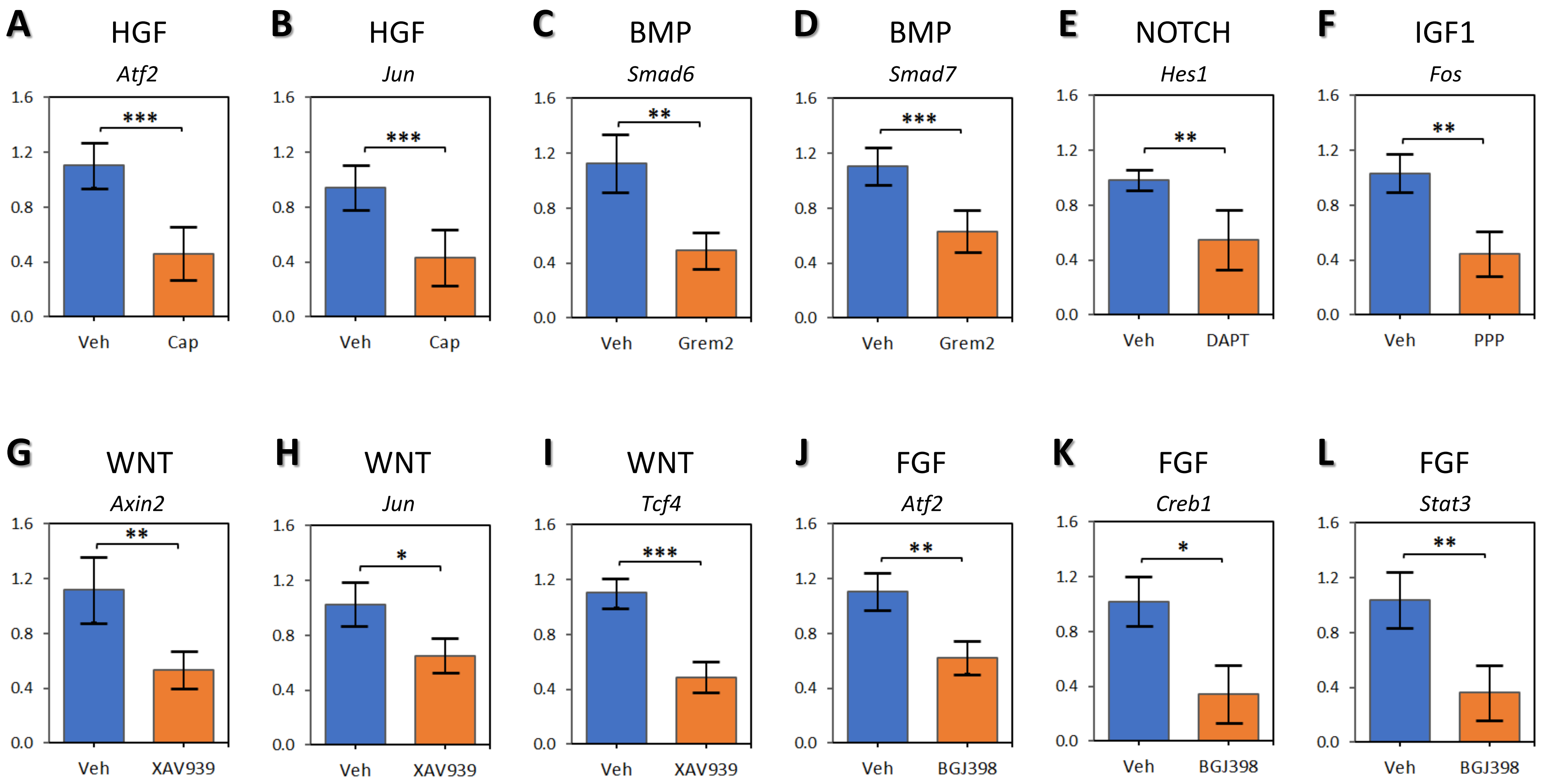

Supplemental Figrue 10: Validation of Inhibitor Function Used in the Organoid Cultures to Block 6 Principal Signaling Pathways in pAT2, Related to Figure 6. To validate the function of each of the inhibitors used in Figure 9, RNA was extracted from control and inhibitor-treated individual organoid cultures (n>=3 for each) and realtime RT-PCR was performed using established downstream targets of each of the 6 principal pathways, HGF (A, B), BMP (C, D), NOTCH (E), IGF1 (F), WNT (G – I) and FGF (J – L). Pathway target genes were selected from Ingenuity pathway report (i.e. https://reports.ingenuity.com/rs/report/cpathway?id=ING%3Acim) and their activity in AT2, was verified by LungMap (www.lungmap.net). Inhibition was confirmed by the reduced expression of the pathway targeted genes selected above. Data are represented as mean +/- SD. A two-tailed Student's T-Test was used for the comparison between the vehicle and the inhibitor treated organoids (* stands for 0.05-0.001, ** for 0.01-0.001, *** for <0.001). For concentration of inhibitors please see Resource Tables in Materials and Methods.

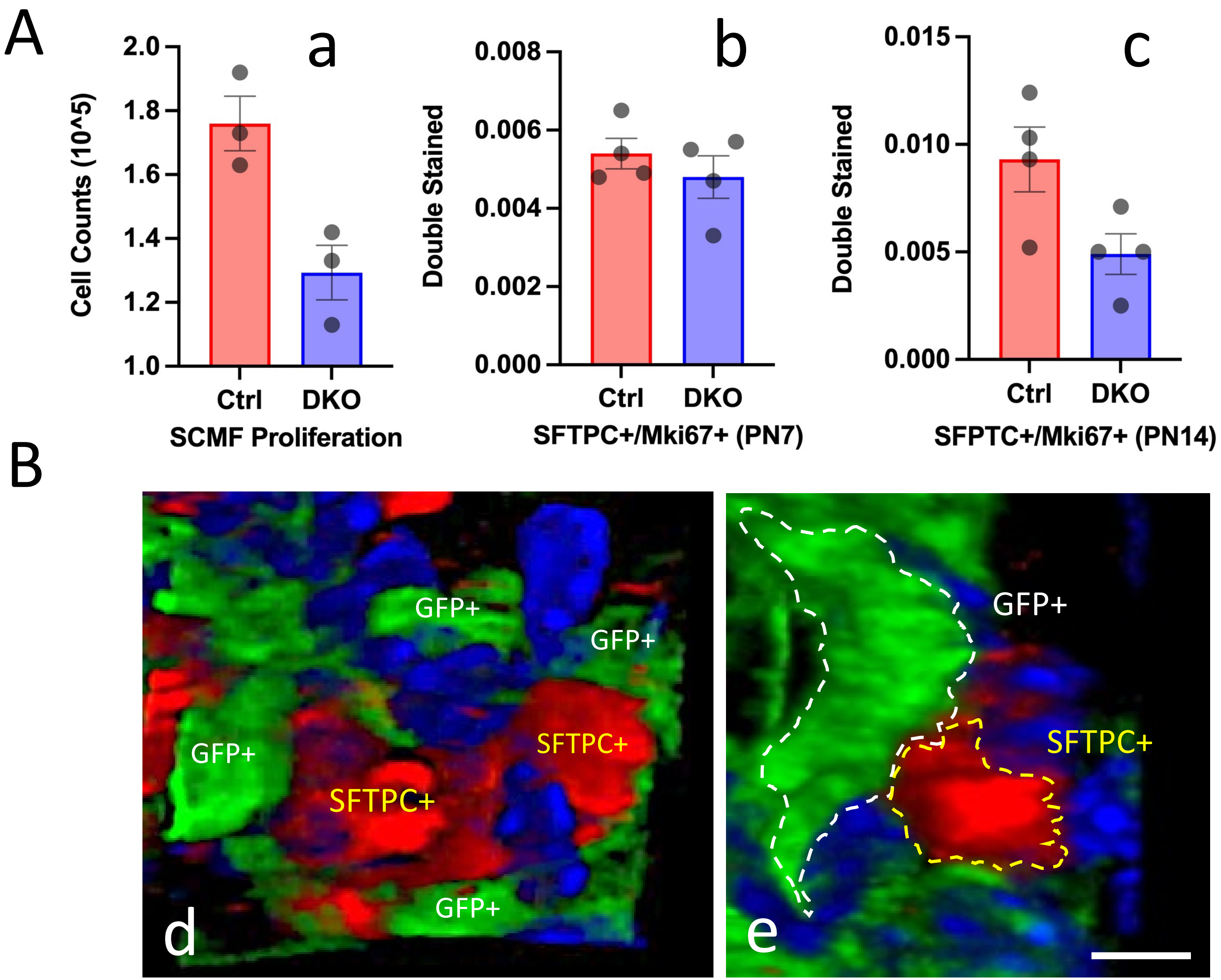

Supplemental Figure 11: (A) Loss of TGFb Signaling to SCMF Reduces Proliferation in SCMF and SFTPCpos Epithelial Cells in PN14 Mutant Lungs, Related to Figure 7. (a) GFP+ SCMF cell lines from mutant and control lungs (n=3 for each) were cultured in vitro for 24 hours and subsequently counted, using hemocytometer. (b & c) Proliferating pAT2 cells were identified as SFPTC/MKi67 double positive cells and scored on PN7 and PN14 control and mutant lung tissue (n=4 biological replicates and n=8 experimental replicates). All values were normalized to total number of cells (i.e. DAPI+) to correct for mutant hypoplasia. Data are represented as mean +/- SD. Ctrl=control, DKO=mutant. (B) Spatial proximity of GLi1-creERT2-labeled (GFP+) fibroblasts to AT2 (SFPTC+) cells in PN14 lung revealed by 3D reconstruction of confocal images in (d) low and high (e) magnifications. Scale bar: 10um for d and 5um for e.

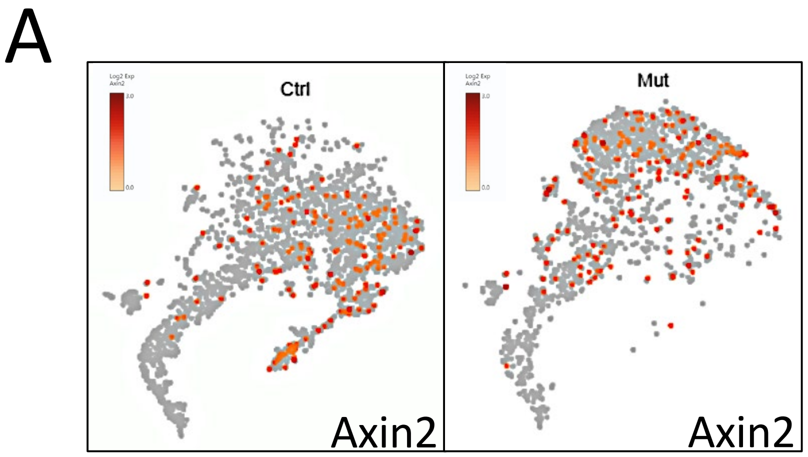

B

Subcluster	CONT	%	MUT	%
C4	29/283	10.2	1/5	20.0
C5	14/225	6.2	8/45	17.8
C6	93/1342	6.9	68/528	12.9
C7	21/395	5.3	88/979	9.0
C8	5/205	2.4	17/149	11.4
C9	6/435	1.4	7/262	2.7
C10	0/125	0.0	0/86	0.0
C11	2/112	1.8	3/46	6.5

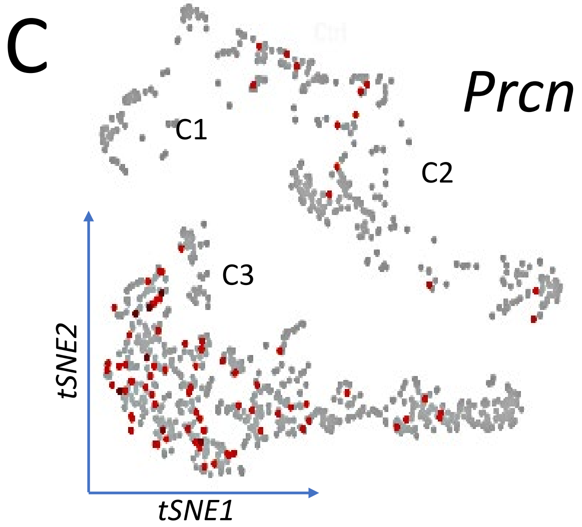

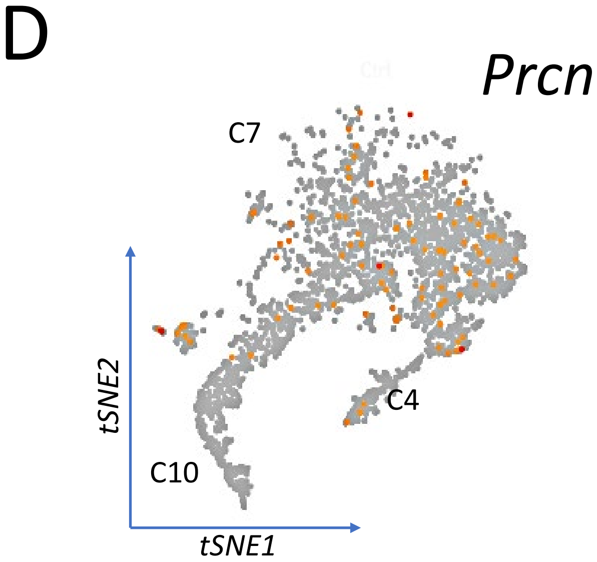

Supplemental Figure 12: Axin2(+) Cells are Scattered throughout the PN14 Epithelial Population and Prcn is Expressed mostly by C3 and not C4 Cells, Related to Figure 7. (A) tSNE representation of Axin2 transcripts distribution in control and mutant EPC. (B) quantification of the number of Axin2(+)/total Sftpc(+) cells and their calculated percentage. (C & D) tSNE representation of Prcn transcripts distribution in MEC versus EPC, respectively.
